# Neuroendocrinological and Epigenetic Mechanisms Subserving Autonomic Imbalance and HPA Dysfunction in the Metabolic Syndrome

**DOI:** 10.3389/fnins.2016.00142

**Published:** 2016-04-14

**Authors:** Erwin Lemche, Oleg S. Chaban, Alexandra V. Lemche

**Affiliations:** ^1^Section of Cognitive Neuropsychiatry, Department of Psychosis Studies, Institute of Psychiatry, Psychology and Neuroscience, King's College London London, UK; ^2^Section of Psychosomatic Medicine, Bogomolets National Medical University Kiev, Ukraine; ^3^Department of Medical Science, Institute of Clinical Research Berlin, Germany

**Keywords:** metabolic syndrome, sympathetic autonomic nervous system, stress neuropsychobiology, hypothalamic-pituitary adrenocortical axis, epigenetic programming, gene regulation, microRNA, pathophysiology

## Abstract

Impact of environmental stress upon pathophysiology of the metabolic syndrome (MetS) has been substantiated by epidemiological, psychophysiological, and endocrinological studies. This review discusses recent advances in the understanding of causative roles of nutritional factors, sympathomedullo-adrenal (SMA) and hypothalamic-pituitary adrenocortical (HPA) axes, and adipose tissue chronic low-grade inflammation processes in MetS. Disturbances in the neuroendocrine systems for leptin, melanocortin, and neuropeptide Y (NPY)/agouti-related protein systems have been found resulting directly in MetS-like conditions. The review identifies candidate risk genes from factors shown critical for the functioning of each of these neuroendocrine signaling cascades. In its meta-analytic part, recent studies in epigenetic modification (histone methylation, acetylation, phosphorylation, ubiquitination) and posttranscriptional gene regulation by microRNAs are evaluated. Several studies suggest modification mechanisms of early life stress (ELS) and diet-induced obesity (DIO) programming in the hypothalamic regions with populations of POMC-expressing neurons. Epigenetic modifications were found in cortisol (here *HSD11B1* expression), melanocortin, leptin, NPY, and adiponectin genes. With respect to adiposity genes, epigenetic modifications were documented for fat mass gene cluster *APOA1/C3/A4/A5*, and the lipolysis gene *LIPE*. With regard to inflammatory, immune and subcellular metabolism, *PPARG, NKBF1, TNFA, TCF7C2*, and those genes expressing cytochrome P450 family enzymes involved in steroidogenesis and in hepatic lipoproteins were documented for epigenetic modifications.

## Causative problems in the metabolic syndrome

Metabolic Syndrome is a consensus construct with wide acceptance based on its clinical usefulness and growing epidemiologic importance. It is currently defined by concurrent appearance of risk ranges in lipid traits, progredient prediabetes, pronounced adiposity with emphasis on abdominal obesity, and subclinical cardiovascular conditions. The concept implicitly assumes that a malignantly degrading spiral increases the probability of a co-existence of pathophysiologically relevant risk ranges, mainly resulting in organ damage resulting from type 2 diabetes mellitus (T2DM), hepatosteatosis, and cardiovascular incidences. Because, in comparison, single MetS components tend to rarely incur alone, plausibility justifies in summary the employment of the MetS construct. It has in the meantime also become clear that the number of metabolic abnormalities is correlated with overall MetS risk, so emphasizing meaningfulness of its usage.

### Acceleration in incidences

In the last decade, several researchers have alarmed the public of an international epidemic of MetS (Zimmet et al., 2001; Ford et al., 2002; Caballero, 2007; Capoulade et al., 2012), which has its greatest acceleration rates in threshold and emerging market countries. MetS is present in only 12.5% of patients with normal glucose tolerance, in 55% of those with impaired fasting glucose, and in 81% of those with T2DM (Ginsberg and Stalenhoef, 2003). The presence of MetS increased the risk of T2DM manifestation 24-fold during a 5-year period (Sattar et al., 2003). Furthermore, CVD risk climbed toward 20% once T2DM had developed in MetS patients (Girman et al., 2004). MetS risk increases with aging, being 44% in the seventh decade of life having the MetS compared to 7% prevalence in the third decade (Ford et al., 2002). There are further implications of MetS for aging biology because of associations of MetS components with oxidative stress (Moreira et al., 2015), DNA damage (Chen et al., 2015), telomere attrition (Révész et al., 2015), and mitochondrial damage due to sirtuin depletion (Guarente, 2006).

### Obesity as a central precondition for MetS

The overall rising incidence of MetS is to a certain degree a consequence of the obesity epidemic in developed and threshold populations. Although obesity figures in the U.S. were historically reported to be on a rise since the first decades of the twentieth century, dramatic accelerations worldwide have been described for the past four decades (Zimmet et al., 2001; Caballero, 2007). According to newest figures announced on 12 October 2015 from the World Obesity Federation, it is suggested that, if current trends continue, 2.7 billion adults worldwide will be overweight by 2025. This is a 35% increase from 2.0 billion in 2014. The developmental precursor of adulthood MetS is seen in childhood ectopic lipid storage interfering with hepatic insulin signal transduction (Nelson and Bremer, 2010; Melka et al., 2013) with similar acceleration rates, and presumably resulting from epigenetic modifications and regulatory programming of obesity genes, and likely induced by nutrition-caused alterations of the gut microbiome (Remely et al., 2014b; Chang and Neu, 2015).

### Brief history: syndrome X to MetS

Over the last 12 years, MetS criteria have seen five major revisions in details; however, all operationalized definitions address specific metabolic abnormalities, hypertension and obesity (Eckel et al., 2010). An early conceptualization of MetS is contained in the *Observationes Medicae* (1641; book II, section 46 on diabetes) of the Amsterdam anatomist Nicolaes Tulp (1593–1674). In the context of gout as diabetic complication spoke French rheumatologist Jean Pierre Camus 1966 of a “metabolic trisyndrome” with hyperlipidaemia. In 1975, Hans Haller of the Dresden Academy of Medicine *Carl Gustav Carus*, coined the term “metabolic syndrome,” unbeknownst to the West, to support his observation of a coincidence of obesity, dyslipidaemia, hepatosteatosis, and disturbed glucose metabolism above chance level (Haller and Hanefeld, 1975; Haller and Leonhardt, 1981). The first description of resistance against insulin-dependent glucose uptake was proclaimed by Gerald Reaven (Reaven, 1988), then named “syndrome X.” Reaven built on clinical observations in the 1920s, of the Swede Eskil Kylin (Kylin, 1921, 1923), the Spaniard Gregorio Marañón (Maranon, 1922), and the American Elliot Joslin (Joslin, 1921). These clinicians had postulated a syndromal cohesion of hypertension with hyperglycaemia in prediabetes, although currently hypertension is no longer seen as a necessary prerequisite for MetS. But the terminology “syndrome X” was soon abandoned, as there were also terms circulating such as cardiac X syndrome and fragile X syndrome. In efforts for clarification, the term was henceforth specified to *metabolic* syndrome X, and then eventually shortened to the Metabolic Syndrome; a current synonym is Reaven-syndrome in commemoration of its modern re-inceptor. The World Health Organization (WHO) defined MetS as a group of risk factors for CVD and T2DM (Alberti and Zimmet, 1998): renaming into MetS was promoted by Eckel et al. (2005); Grundy et al. (2005a,b); Turek et al. (2005), with new guidelines 2005 and 2006 of the American Heart Association and the International Diabetes Federation, respectively, representing current standards.

### The quest for causation

**Figure 1:**
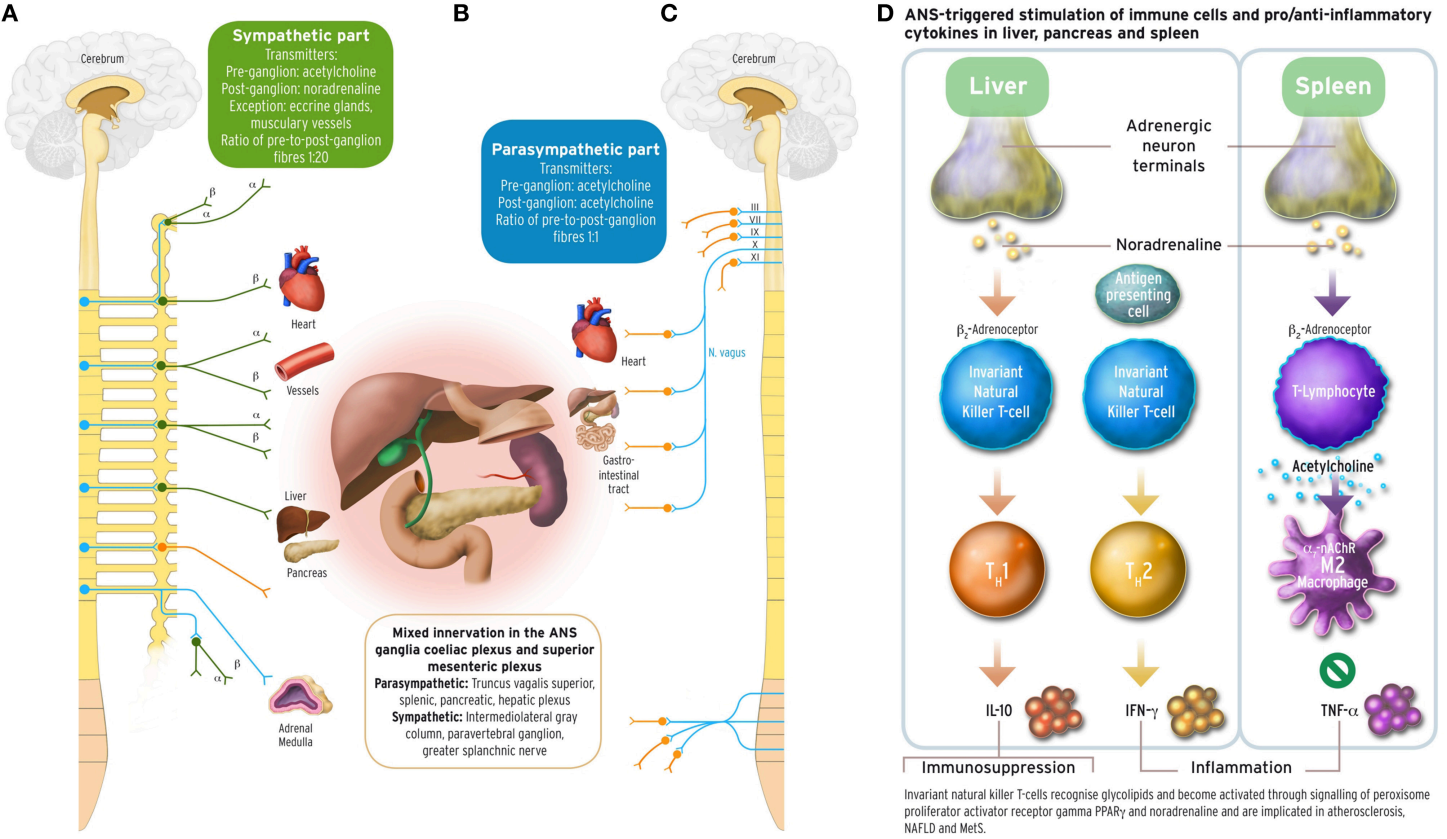
**Sympathetic and parasympathetic innervation of the coeliac and superior mesenteric plexus ganglia, and immune and cytokine mechanisms in cholinergic anti-inflammatory pathway**. The acetylcholinergic anti-inflammatory pathway, the efferent arc of the inflammatory reflex (Section Inflammation and Arterial Rigidity; Tracey, 2002) converging in the spleen, has been discussed under the aspect of being a target for possible interventions counteracting autonomic imbalance in the metabolic syndrome related to chronic inflammation. Schematically depicted are innervations from the sympathetic and parasympathetic branches of the ANS, with their transmitters, into organ systems relevant for MetS. Left part panel **(A)**: efferent fibers in the sympathetic branch with adrenoceptor type. Middle part panel **(B)**: mixed sympathetic and vagal fiber connections into the coeliac and the superior mesenteric plexus ganglia innervating liver, pancreas and spleen. Right part panel **(C)**: efferent fibers in the parasympathetic branch of the ANS. The insert panel **(D)** to the outer right hand side illustrates schematically the role of vagus stimulation-derived, yet noradrenergic transmission into liver and spleen, and the acetylcholinergic transmission between CD4^+^ T helper cells and macrophages in the spleen. Vagus departs the brainstem as its cranial nerve X, and vagal efferent outflow regulates visceral organs by counterbalancing sympathoexcitation, inhibiting cytokine release, and safeguarding against inflammatory damage to liver, pancreas, spleen, lungs, or kidneys in endotoxaemic states. The outflow of the vagus nerve triggers adrenergic neurons in the coeliac ganglion innervating the spleen further to liver and pancreas. Vagal influence to spleen T lymphocytes stimulates the release of the neurotransmitter acetylcholine (ACh), and activation of the α_7_ subunit of the nicotinic ACh receptor (α_7_ nAChR; Section Inflammation and Arterial Rigidity) expressed on cell membranes of splenic macrophages and other cytokine secreting cells. Vagal tone attenuates here production of the inflammatory response cytokine tumor necrosis factor alpha (TNFα) reciprocally related to sympathoexcitation (Zhang et al., 2003; Kisiswa et al., 2013). In the liver, noradrenergic innervation signals hepatic innate natural killer T cells (iNKT) (Van Kaer et al., 2013) to exert systemic immunosuppression. Increasing the vagal tone there induces a shift from pro-inflammatory T helper cell type 1 (T_H_1) cytokines such as interferon-γ (IFN-γ) to anti-inflammatory T_H_2-type cytokines, such as interleukin-10 (IL-10) (Tracey, 2007, 2009; Rosas-Ballina et al., 2008, 2011; Trakhtenberg and Goldberg, 2011). PVNH hypothalamic insulin promoter expressing neurons downregulate postprandial inflammation through cholinergic signaling in the spleen mediated by vagal outflow to the spleen, whereas vagotomy results in T2DM (Carvalheira et al., 2014; Wang L. et al., 2014). Vagus stimulation approaches for increasing vagal tone would therefore aim to counterbalance prolonged sympathoexcitation in MetS by supporting parasympathetic output. These could comprise, but are not limited to, device-based, pharmacological, and/or psychotherapeutic intervention approaches. With the advent of wearable transcutaneous stimulation devices, vagus nerve stimulation has become a convenient neuropsychological intervention method (Van Leusden et al., 2015). Drug discovery is still required to identify non-steroidal anti-inflammatory substances targeting the cholinergic pathway either peripherally (such as nicotinic α_7_ nAChR agonist applications) and/or centrally (such as CNI-1493), or existing TNFα antagonists such as *infliximab* or *etanercept*. Possible behavioral interventions: Guided physical activity trainings, mindfulness-based psychotherapies, psychosomatic body-relaxation and/or balancing techniques, biofeedback training. Other: immunotherapy yet to be developed (Van Kaer et al., 2013). Medical illustrations by Corinna Naujok, Charité Media Centre Berlin, Virchow Campus.

Abnormalities in the anterior pituitary gland and other hypothalamic structures regulating hunger-satiety homeostasis through the polypeptides leptin and ghrelin (Turek et al., 2005), and the melanocortins MSH and ACTH (Iwen et al., 2008), are considered responsible for MetS. These lead to defects in the hypothalamic-pituitary-adrenal axis (HPA), which may progress to the onset of T2DM. Recent evidence suggests that imbalanced autonomic nervous system output causes the simultaneous occurrence of T2DM, dyslipidaemia, hypertension, and visceral obesity: MC4R neurons in amygdala, arcuate nucleus, paraventricular nucleus, nucleus suprachiasmaticus, and anterior pituitary regulate food intake (Turek et al., 2005; Buijs and Kreier, 2006), energy expenditure (Balthasar et al., 2005) or influence vasoconstriction via angiotensin mediated activity of the sympathetic nervous system (Greenfield et al., 2009). There is also evidence that hepatic cholesterol reuptake is steered through parasympathetic pathways by MC4R-expressing neurons (Perez-Tilve et al., 2010; Krashes et al., 2016). Of endocrinologic factors, elevated cortisol levels are suspected to contribute to insulin resistance (Lewis et al., 2010). Such associations between sympathetic hyperexcitability, HPA axis hyperactivation, and decreased vagally mediated (anti-)inflammatory reflex (Figure 1), and MetS features were found in T2DM sufferers, where elevated plasma cortisol predicts greater prevalence of CVD (Reynolds et al., 2010).

## Traditional hypotheses on aetiopathology

### Malnutritional factors

Because genetic susceptibility varies strongly amongst different ethnicities, and because the overall gene pool did not substantially change over the last decades, the obesity and diabetes epidemic was assumed resulting mainly from detrimental environment factors, such as food availabilities, diet habits, or sedentary lifestyles.

#### Carbohydrate nutritional factors

Several studies have described a nutritional dependence of MetS features such as hypertriglyceridaemia and other lipid traits (primarily low-density lipoprotein, LDL), and insulin secretion (Lofgren et al., 2005; Volek and Feinman, 2005; Volek et al., 2005; Forsythe et al., 2008). Caloric restriction exerted by means of very low carbohydrate diets also reduces a range of pro-inflammatory markers when a reduction in adipose tissue can be reached. In reverse direction, high-calorie diet is inductive of obesity within a one-week span, and also correlated with elevated sympathetic activity (da Silva et al., 2014). It has, from this perspective, been argued that MetS manifestation is a consequence of high carbohydrate intake, as it attenuates high fasting glucose levels, triggers insulin secretion, contributes to high plasma triglycerides, attenuates the HDL proportion and supports high blood pressure (Volek and Feinman, 2005; Westman et al., 2007). A further transitional state toward MetS is seen in the development of a fatty liver disease (NAFLD), including dyslipidaemia, associated with sedentary life style and lack of physical exercise (Pinto et al., 2015). High carbohydrate intake is typically associated with microbiotic fermentation of nondigestible polysaccharides, which are transformed into better manageable short chain fatty acids (SFCAs, acetate, propionate, and butyrate) that, however, increase reactive oxygen species (ROS), pro-inflammatory cytokines, decrease gut membrane integrity, and induce epigenetic modifications via inhibition of histone deacetylases, thus reducing respective gene expression (Tan et al., 2014).

#### Fatty diet consumption and fat uptake

Another theoretical approach assigns priority to evidence stating fatty diet in first place rather than carbohydrates, although there is on-going controversy as to which of the two is more detrimental. Animal evidence suggests that consumption of saturated fat and high-cholesterol diet resembling animal fats is able to induce NAFLD, consistent with hepatic fat accumulation and inflammation (non-alcoholic steatohepatitis, NASH) as manifestation of MetS (Pavlov and Tracey, 2012; Mells et al., 2015). Experimental results suggest that cholesterol is correlated with leptin, interleukin-6 (IL-6), liver weight and liver weight/body weight ratio, fibrosis and α-smooth muscle actin (α-SMA) (a marker of myofibroblast formation) in the liver. The alternative view (Mells et al., 2015) argues that a lower carbohydrate and higher fat diet is more favorable in terms of reduction of abdominal and intramuscular fat deposition and the prevention of T2DM. Intervention studies relate this to more favorable fasting insulin and glucose levels, as well as activation of pancreatic β-cells.

According to novel theoretical accounts and findings (Parekh et al., 2014; Grundy, 2015), play SFCAs fat metabolites (SFCAs, short chain fatty acids, acetate, propionate, and butyrate) produced by the gut microbiota a key role in arriving at insulin resistance and obesity. These SFCAs may better serve increase in energy expenditure, but at the cost of greater lipid storage. SFCAs affect satiety sensing, and contribute to downregulating neuropeptide Y and the glucagon-like peptides with the consequence of hyperphagia. According to this theorizing enter SFCAs more easily colonic epithelium, interact with high caloric nutrients with the consequence of epigenetic programming. Furthermore, this may lead to a bias of the immune system by activation of T cells in the gastrointestinal tract, their migration into adipose tissue, and to maintenance of low-level inflammation (Chang and Neu, 2015). The evaluation of existing research findings also suggests that dysbiosis due to high-fat diet and reduction of gut biodiversity increases permeability of the intestine (Winer et al., 2016), with the consequences of systemic circulation of gut hormones and pro-inflammatory cytokines, and of reduction of proper functioning of mesenteric lymph nodes.

#### Sodium and natriuresis

Salt sensitive regulation of blood pressure is documented in animal and human studies (He et al., 2013), and more pronounced in specific populations in accordance with genotype and environmental interactions (Sanders, 2009). Specifically, genes regulating renal function toward sodium excretion with the cytochrome enzyme class P450 (*CYP11B2, CYP4A11, CYP3A5*, see also Section Obesity: Lipid Transport and Storage) via aldosterone synthesis, and dopamine-receptor-1-mediated salt excretion, have been associated to blood pressure lowering in hypertensive rodents. Salt consumption and sodium levels related to essential hypertension have been substantiated for several decades (Svetkey et al., 1987; Deter et al., 1996, 2002, 2007a,b; Buchholz et al., 1999), and been found to be influenced by emotional arousal (anger, anxiety) involving sympathetic outflow. A recent epidemiological study quantified the explained variance for trait anxiety upon high blood pressure to 6% (Lemche et al., 2016) in MetS. Analyses of population study databases (Oh et al., 2015) confirmed a linear dependence of sodium excretion, blood pressure measures, glucose and insulin levels, lipid traits, and several fat mass measures in MetS patients, even in the absence of clinical hypertension, thus making it a new nutritional risk factor of MetS. High sodium intake showed positive linear association with glucocorticoid secretion and metabolites, insulin resistance, and inverse association with adiponectin (Baudrand et al., 2014).

Whereas, nutrition-related risk factors are critical for all the metabolic causations (Szczepanska-Sadowska et al., 2010; Thorp and Schlaich, 2015) nominated for MetS in recent theoretical concepts on MetS, namely (a) overeating, (b) insulin resistance, (c) visceral adiposity, (d) arterial stiffness, for the one non-metabolic, namely (e) chronic stress, mechanisms are located in central and autonomic nervous systems.

### Stress-related factors

Although stress has been implicated in the pathophysiology of MetS for decades (Brindley, 1995; Peeke and Chrousos, 1995), the best-regarded epidemiological evidence on this risk factor is commonly attributed to the Whitehall II study. Whitehall II is a prospective cohort study of >10 k British civil servants, which are being investigated stratified for employment grade and including diurnal cortisol probes. The overall finding from the stratified results Brunner et al. (2002); Chandola et al. (2006, 2008) is that psychological stress measures accounted for 37% explained variance of the correlation between MetS and normetanephrine, heart-rate variability, cortisol and interleukin-6, whereas health behaviors explained 18% in neuroendocrine responses. Cross-sectionally, there was a relation of work stress to cortisol secretion, and longitudinally, a dose-response relation between chronic stress and MetS.

The first reports on MetS as long-term sequela of posttraumatic stress disorder (PTSD) appeared in context of paediatrics and childcare. Reports investigating drug abuse in veterans stated strong obesity figures, hypertension, T2DM and dyslipidaemia, which were not attributable to drug consumption. Three recent meta-analyses (Edmondson et al., 2012; Bartoli et al., 2013; Rosenbaum et al., 2015) confirmed an increased risk (ORs 1.6–2.0) for MetS and CVD in PTSD sufferers, with 35–50% prevalence of single MetS components (whereby only 20% expectable). The occurrence of MetS was further related to accelerate T2DM with accelerated age-related cognitive decline in veterans suffering from PTSD (Green E. et al., 2015).

#### Vagal-sympathetic imbalance

There is increasing evidence for autonomic imbalance in MetS, the exact nature of this sympathetic hyperexcitability appearing to have primarily consequences for the development of obesity and insulin resistance. Known are age differences in the balance of vagal and sympathetic cardiac and vasomotor regulation (Hart et al., 2014), resulting in lesser sympathetic activity in elderly, which would thence bias toward prediabetes by increasing age. Blunting of SNS outflow toward glucose intake is a characteristic response of diabetic MetS sufferers (Straznicky et al., 2009). On the other hand, it is a replicated finding that the “vagal brake,” namely heart rate recovery after arousal, is absent in MetS, and that this prolonged sympathoexcitation is also associated with single MetS components (Deniz et al., 2007; Kim et al., 2009). Evidence for chronic sympathoexcitation in MetS consists in (a) elevated urinary and plasma noradrenaline levels, (b) enhanced efferent muscle nerve activity, but not necessarily essential hypertension (Thorp and Schlaich, 2015). SNS hyperreactivity decreases muscular blood flow and glucose uptake thus leading into insulin resistance. Heightened sympathetic outflow further acts toward β-adrenoceptor sensitization. Microneurography of postganglionic sympathetic responses in firing bursts is here the best feasible measurement. Consistently replicated evidence shows that peripheral sympathetic muscular firing rates in MetS are related to its obesity component (Grassi et al., 2005; Straznicky et al., 2008). Efferent sympathetic traffic correlated with abdominal obesity and was inversely correlated with baroreflex functioning. SNS muscular neurography rates covaried with plasma noradrenalin; this is in contrast to cardiac sympathetic components (Mancia et al., 1998), which are less so. However, it has been shown that high levels of fasting insulin, an index of insulin resistance, were positively associated with the low-to-high frequency (LF/HF) ratio of the heart rate variability (HRV)—an index of the sympathovagal balance at the heart level (Emdin et al., 2001). Recent experimentation has demonstrated that simple vagotomy leads quickly into insulin insensitivity and T2DM (Wang L. et al., 2014). Insulin promoter neurons in the paraventricular, arcuate, dorsomedial, ventromedial, and lateral hypothalamic nuclei signal to the phosphoinositide 3-kinase (PI3K)/mTOR pathway (Richard, 2015) critical for cell survival to exert control upon the vagus through efferent neurons in the nucleus tractus solitarius and dorsal motor root nucleus of vagus, ultimately terminating in the spleen (Wang L. et al., 2014).

#### Effects of sympathetic outflow

In the meantime have a number of studies also suggested that development of insulin resistance may be induced by sympathetic activity (Masuo et al., 1997; Julius et al., 2000; Esler et al., 2006; Rafiq et al., 2015) based on associated TNFα contribution (Pavlov and Tracey, 2012). However, a correlation between insulin secretion and peripheral sympathetic activity is not generally present (Masuo et al., 1997; Curry et al., 2014), specifically not in young healthy individuals. It may thus be concluded that additional factors, such as nutritional state and/or age range (see above), may be required to develop insulin resistance. It is also possible that insulin resistance and hyperinsulinaemia develop as consequence of an obesity-SNS interaction that then triggers elevated adipokine levels (Thorp and Schlaich, 2015) inducing T2DM (see Section Melanocortin Receptors and Binding Sites).

Current models of sympathetic effects on renal functions assume (Rafiq et al., 2015) that SNS hyperarousal leads to elevated plasma noradrenalin release levels and renal upregulation of glucose transporters (which then trigger insulin secretion from pancreatic β-cells). When tissue, skeletal, and hepatic glucose uptake decreases, blood glucose level and plasma insulin increase, and result in insulin resistance. In this model, SNS induces T2DM by hypertension, but it may therefore not suffice for hyperinsulinaemia in MetS. Essential hypertension may simply be a consequence of preferential fat mass accumulation around kidneys, thus physically interrupting normal functioning of the renin-angiotensin-aldosterone system (RAAS) (da Silva et al., 2014), with the consequence of diminished renal-pressure natriuresis. Typically, however, there is a certain interrelation between obesity and hypertension (da Silva et al., 2014), although in MetS, hypertension may be absent, and vice versa. Also, in many instances, obesity, and hypertension present as non-covarying, thus suggesting partly distinct pathophysiological mechanisms. Repeatedly have physiological parameters obesity and hypertension (Lemche et al., 2016) in MetS samples formed distinct latent factor-analytical clusters. Not peripheral SNS activation leading to general vasoconstriction, but specific renal-sympathetic effects on the RAAS involving sodium retention is experimentally documented to result in essential hypertension (da Silva et al., 2014).

#### Catecholamine system-related

In 1986, Landsberg (Kaufman et al., 1986; Landsberg, 1986; Landsberg and Young, 1986) first described an association of sympathetic outflow and noradrenaline levels, and carbohydrate (but not protein) intake (Kaufman et al., 1986; Landsberg, 1986; Landsberg and Young, 1986), with subsequent insulin action and thermogenesis, finally resulting in hypertension. With respect to hypertension, angiotensin II release was found related to noradrenergic function (Taddei and Grassi, 2005). Because catecholamine release is directly related to SNS hyperreactivity (Section Malnutritional Factors), these two factors are usually considered as one system, primarily mediating short-term stress response [also termed sympathomedullo-adrenal (SMA) axis].

#### Glucocorticoid system related

Cortisol secretion, in contrast, is a physiological response to cope with repeated anxiety triggers as a result of chronic stress (Brown et al., 1982; Fisher et al., 1982; Rivier et al., 1982). Because of striking metabolic parallelisms of MetS and Cushing's Syndrome, the latter is generally considered a clinical model disease for MetS (Anagnostis et al., 2009). While hypercortisolism was early suspected to be causative for MetS (Brindley, 1995; Peeke and Chrousos, 1995), first diachronic evidence that chronic stress is largest cause of MetS via HPA and glucagon upregulation (Brunner et al., 2002; Wang, 2005) was documented in the Whitehall II sample of middle aged (Anagnostis et al., 2009). In higher ages, then, there is direct synchronic association between cortisol levels and MetS criteria (Vogelzangs et al., 2007; Almadi et al., 2013). Whilst attempts to linking MetS components with diurnal cortisol secretion were not always successful (Abraham et al., 2013) (for methodological or endocrine reasons), controversies have finally been resolved by the corroboration that the extent of hair cortisone deposition is related to abdominal obesity and other MetS components (Stalder et al., 2013; Kuehl et al., 2015).

### Inflammation and arterial rigidity

Originally, adiposity was assumed to be the mere cause of MetS (and hence MetS but the exaggeration of obesity), but it is a relatively novel notion (Grundy, 2015) that it is rather the array of agents secreted by white adipose tissue, amongst them adipokines and cytokines, inducing chronic latent inflammation in MetS and obesity (Tilg and Moschen, 2006; Pavlov and Tracey, 2012). Adipokines consisting of leptin, adiponectin and others, pro-inflammatory cytokines (ILs1~18, tumor-necrosis factor-α TNFα), metabolic factors (resistin, visfatin, adipocyte fatty acid binding protein 1, apolipoprotein E), acute phase and immune proteins (CD40, CD40L, C-reactive protein CRP, serum amyloid A3, plasminogen activator inhibitor-1 PAI-1, macrophage migration inhibitory protein 1 MIP1), several angiogenic and endothelial growth factors, and angiotensinogen (Ali et al., 2013). Adipokines influence satiety sensation and pancreatic insulin responses, while cytokines introduce a pro-inflammatory, and pro-thrombotic state with elevated levels of CRP, TNFα, and IL-6 regulated by immune transcription protein NF-κB activation (Jimenez-Gomez et al., 2013) of dependent cytokine gene loci. Nuclear factor NF-κB also is, along with other factors (IL-6, cytokine signaling suppressor proteins, and endoplasmic reticular oxidative stress) (Tilg and Moschen, 2006), implicated in inflammation-induced T2DM (Cyphert et al., 2015), however, its own two gene loci (*NFKB1* 4q24 and *NFKB2* 10q24) have not been found associated with MetS themselves. NF-κB activation is though indirectly crucial for mediation of leptin and insulin effects on hypothalamic *POMC* expression (Plagemann et al., 2009). Postprandial inflammatory and immune responses (particularly pronounced toward high-fat and sucrose diets) are currently seen as a normal transitional state during digestion, and hence the Metabolic Syndrome as an exaggerated and enduring postprandial inflammation state (Pavlov and Tracey, 2012). Clinical evidence suggests that increased inflammation markers could more closely be associated with MetS components (as seen in atypically depressed) than with dysregulated HPA axis and higher cortisol levels (as seen in melancholy) (Lamers et al., 2013). Pro-inflammatory markers CRP and IL-6 are associated with total bodily fat mass impairing physical performance in higher ages (Beavers et al., 2013). Inflammation marker CRP is also the mediatory variable linking MetS with later age-related reduction of cognitive capacities (Dik et al., 2007).

#### Excitation of inflammatory response

The exact mechanism of chronic low-level inflammation induced by action of adipose tissue is based on T-cell and macrophage accumulation (with expression of key adipogenic factors, PPARγ, CCAA-enhancer-binding protein C/ebpβ, insulin-like growth factor IGF, and toll-like receptors 2 and 4 TLR2/4s) in adipocytes (Sun et al., 2012; Ali et al., 2013; Lefterova et al., 2014), and a lack of vagally mediated downregulation of the “inflammatory response” in MetS (Pavlov and Tracey, 2012; Figure 1). Hepatic afferent stimulation of the vagus nerve via the nucleus tractus solitarius excites efferent vagal outflow counteracting excessive cytokine production and thus ameliorating inflammatory responses. This limiting mechanism to the inflammatory response, the (anti-)“inflammatory reflex,” requires the expression of α_7_ nicotinic acetylcholine receptors (α_7_ nAChR), a ligand-gated ion channel expressed on macrophages, lymphocytes, and neurons, in the cholinergic signaling pathway (Olofsson et al., 2012). Obesity is further characterized by decreased energy expenditure, and heightened food intake, while high levels of leptin are released (Barnes and McDougal, 2014). In this context, also SNS activation is involved: Adipose tissue is only innervated by sympathetic nerves (Thorp and Schlaich, 2015), and lipid storage (uptake of fatty acids as triacylglycerides) is also dependent of binding of catecholamines and the pancreatic hormone glucagon to β-adrenoceptors on the surface of adipocytes, thereby activating adenylate cyclase (resulting in cAMP intracellular signaling; Ali et al., 2013). Accumulation of white adipose tissue in obesity is accompanied with indicators of inflammation IL-6 and CRP, but recent evidence suggests that (a) muscle sympathetic activity, and (b) vasomotor activity influencing hypertension are independent of tissue inflammation (Barnes et al., 2014). However, there seems to be a specific interaction of pro-inflammatory cytokine CRP and cortisol in MetS, leading to inhibition of lipoprotein lipase activity and concentration of nonesterified fatty acids (NEFAs) (Perry et al., 2001) in adipose tissue.

#### Inhibition of inflammatory response: The anti-inflammatory reflex

It has been found that agouti-related protein (AgRP, Section Neuropeptide Y and Agouti-Related Hormone Receptors and Binding Sites) stimulates the HPA axis to release ACTH, cortisol, and ACTH in response to IL-1β in adipose tissue (Xiao et al., 2003), suggesting that elevated cortisol secretion in MetS could be inhibitive to adipose tissue inflammation. It is yet unclear, however, whether this is a mechanism that could replace or override the vagally mediated cholinergic signaling as part of the efferent branch of the anti-inflammatory reflex arc (Figure 1 and above). This would then be a reinforcing effect of plasma cortisol as part of the inflammatory response, in which TNFα and sympathoexcitation are reciprocal (Zhang et al., 2003). However, there is novel evidence also suggesting a secondary CRH system outside the HPA in adipose tissue (Section Adiponectin and Genomic Bases) (Seres et al., 2004; Fahlbusch et al., 2012; Subbannayya et al., 2013; Dermitzaki et al., 2014).

#### Inflammatory and oxidative stress related to CVD

Increased inflammatory markers are also a concomitant of insulin resistance onset (Moreira et al., 2015) induced with adiponectin, resistin, and TNFα. Leptin also induces the production of nitric-oxide synthase 2 (NOS2) and, thereby, ROS. Angiotensin II (see below) and superoxide or ROS are amongst the main endothelium-derived constriction factors (Kang, 2014; Young and Davisson, 2015). It is hereby assumed that hyperoxigenation of LDL is the source of endothelial plaque genesis. ROS generation is assumed being caused by excess nutrient processing in mitochondria, triggering even further ROS accumulation, and impairing endoplasmic reticulum (ER) function in protein folding (Hotamisligil, 2010a,b; Hummasti and Hotamisligil, 2010). Vagally induced relaxation of endothelium occurs by acetylcholine signaling (Figure 1) and is critical for release of the endothelium-derived hyperpolarizing factor (EDHF). Normal regulation of vascular tone is increasingly impaired by reduction of EDHFs leading to endothelial dysfunction associated with CVD (Young et al., 2015).

#### Endothelial dysfunction and angiotensin II release

In addition to the immunological and inflammatory factors detailed above, adipose tissue produces also the peptide angiotensin II, a regulator of hydrolysis in the RAAS via hypothalamic AT_1_-receptors. In blood vessels, AT_1_-receptors induce vasoconstriction, and thus may promote thrombosis and vascular injury (Perry et al., 2001). Increasing imbalance between vasoconstrictors and vasodilators or relaxants (Moreira et al., 2015) then causes impairment of baroreflex functioning through atherosclerosis. Impairment of baroreflex functioning is considered being a main cause of MetS, although hypertension is not essentially necessary for MetS. Hypothalamic arcuate nucleus is the regulatory site for insulin and lumbar baroreflex action (Cassaglia et al., 2011), and efferents via the paraventricular nucleus pathway.

## Genomic foundation of basic regulatory mechanisms in MetS

### Obesity: Lipid transport and storage

With both obesity and hyperlipidaemia are two MetS features related to deposition and metabolism of lipids. There is accumulating evidence (Farmer et al., 2008; Rivera et al., 2012; Cole et al., 2014) that genetic and epigenomic mechanisms steering lipid transport, uptake, and catalysis are central to MetS pathophysiology. In this general context, mediation of SNS for lipid transport and storage is present, as α_2_-adrenoceptors inhibit lipolysis, and β_2∕3_-adrenoceptors trigger lipolysis in adipose tissue, in addition to regulatory functions for glucagon, insulin, renin, and ghrelin secretion.

#### Genomic loci of plasma lipid traits

Plasma lipid traits are regulated by genomic loci interacting. One hundred and eighty five common variants were described in two large-scale studies of the Global Lipids Consortium (Teslovich et al., 2010; Do et al., 2013), amongst them *APOA1, APOB, APOE*, and *TRIB1*. Many SNPs are in close vicinity to those 18 loci known to cause Mendelian lipid disorders, such as the *TRIBAL* locus downstream from *TRIB1*, and exhibiting reciprocal regulatory expression (Proudfoot, 2011; Douvris et al., 2014). Within these apolipoprotein genes, specific genetic factors for MetS (i.e., SNPs relevant for MetS risk) became evident in the Diabetic Heart Study (Adams et al., 2014), and others (Crosby et al., 2014; Gaio et al., 2014): *rs*3135506 (Ser19Trp, APOA5), *rs*651821 (5′UTR, *APOA5*), *rs*13832449 (splice donor, *APOC3*) (Crosby et al., 2014). The *APOA5* on locus 11q23, has binding affinity with LDL-Receptor gene (Nilsson et al., 2008), and the *APOA5*-related *HTG* specifically risky for CHD (Zhou et al., 2013). *APOA5, APOC3, APOA1*, and *APOA4* loci are closely clustered in 11q23 (Nilsson et al., 2008), with SNPs *rs*2972146 near *IRS1* locus shown associated with increased risk of T2DM, insulin resistance and hyperinsulinaemia (Teslovich et al., 2010); *rs*1042034 of *APOB* related to HTG as main lipoprotein of chylomicrons and lipid-rich particles (Teslovich et al., 2010), located on chromosome 2. *APOC3* is related to VLDL, and affects lipid levels by postponing triglyceride catabolism (Russo et al., 2001). In reverse: a missense mutation lowers plasma triglycerides and CVD risk (Crosby et al., 2014). *APOA-V* suppresses exuberance of triacylglycerides (Pennacchio et al., 2001) by creating a feedback-loop for downregulation of apolipoprotein A5 (Caussy et al., 2014).

#### Genomic loci for fat mass accumulation

The European multicentre study of Aulchenko had shown that further to cholesterol/lipoprotein metabolism also lipid transport/obesity is a second cluster in CVD risks. The major locus amongst lipolysis genes is the *LIPE* (19q13.2) gene encoding lipase, where carriers of the D-allele show distortion in lipid metabolism and insulin sensitivity (Albert et al., 2014), also in CYP2C19, a monooxydase protein of the cytochrome P450 family, involved in catalysis of lipids, cholesterol and other steroid hormones such as cortisol (Gaio et al., 2014). It is possible that, in the MetS constellation, additional risk genes are relevant, such as *CYP2C19* (10q24) (Gaio et al., 2014). The major lipid storage genes are those for the peroxisome proliferator-activated receptor γ (*PPARG* 3p25) mainly found in adipose tissue (Gu et al., 2014), and furthermore the obesity-related *FTO* (16q12.2) (Yang et al., 2014). A common variant of the latter *rs*9939609 has been found relevant for MetS components, hypertension, dyslipidaemia and CVD (He et al., 2014; Liguori et al., 2014). Common and rare variants in the melanocortin-4 receptor *MC4R* locus 18q21.32 (*rs*74861148, *rs*483125, and *rs*11872992) or its promoter region were associated with triglycerides, obesity and T2DM (Bazzi et al., 2014; Katsuura-Kamano et al., 2014; Muller et al., 2014).

### T2DM: Melanocortins and insulin resistance

Two hundred and twenty one million individuals were suffering from T2DM in 2010 worldwide, and expected are 300 million sufferers by the year 2025 (Zimmet et al., 2001). Regarding the heritability of T2DM, it is currently conceived that gene polymorphisms pertaining to obesity, insulin resistance, dyslipidaemia, glucose uptake and pancreatic β–cell dysfunction coact in a way that ultimately results in T2DM (Ridderstråle and Groop, 2009). Genetic susceptibility is a necessary prerequisite to develop T2DM: in monozygotic twins there is 70% concordance, and in parent-offspring there is a risk of more than 40% probability (Lyssenko et al., 2005). T2DM is both a monogenic and a polygenic condition with a multitude of genes involved (Stumvoll et al., 2005). Eighteen T2DM gene loci have been isolated in genome wide scans (GWAS), the best replicated of which are melanocortin receptor-4 (*MC4R*), T-cell factor 7-like 2 (*TCF7L2*), and peroxisome proliferator-activated receptor gamma (*PPARG*) (Stumvoll et al., 2005; Ridderstråle and Groop, 2009) genes, but each gene with relatively small effect size (ORs 0.8–1.3).

#### Characterization of functions

In several populations has the *TCF7L2* (10q25.3) locus shown association with T2DM (Assmann et al., 2014; Ouhaibi-Djellouli et al., 2014). This locus encodes a transcription factor implicated in blood glucose homeostasis. It also regulates total cholesterol, LDL and HDL, and was found contributing to MetS in the context of increased CHD mortality (Khoroshinina et al., 2014). Peroxisomes are normally small cytoplasmic vesicles oxidizing by their enzymes fatty acids, and are also related to glucose metabolism. The PPAR nuclear receptors, which include PPARα, PPARδ, but particularly PPARγ, are transcription factors that mediate effects of fatty acids and their derivatives on gene expression, are, together with the mutually co-expressed C/ebpβ, the “master regulators” of adipogenesis (Lefterova et al., 2014). The protein encoded by *PPARG* (3p25) regulates adipocyte differentiation (Ali et al., 2013). All three PPARs, but especially PPARγ, are expressed in macrophages and modulate adipose tissue inflammation. *PPARG* was associated with T2DM in several populations (Black et al., 2015; Katome et al., 2015). Specifically the MC4R, less so MC3R, is expressed in the CNS widely (Millington, 2007) and related to feeding (and other) behaviors. Defects in the *MC4R* (18q22) lead to infantile hyperphagia, childhood obesity, elevated plasma insulin levels, and growth acceleration. The *MC4R* was alone or in combination with the *FTO* gene related to T2DM and food intake (Huang et al., 2011; Statsenko et al., 2013), and CVD. As the MC4R is capable to bind to a larger array of melanocyte stimulating hormones (MSHs), or melanotropins, specific mechanisms of action lie in the different ligands and probably cerebral locations. MSHs are a group of peptide hormones synthesized in hypothalamus and pituitary, binding to the same group of melanocortin receptors (MC1-5Rs). Melanotropins modulate central energy expenditure and regulate hunger feelings. From the proprotein pro-opio-melanocortin (POMC), α-MSH, β-MSH, and two γ-MSH isoforms are derived by cleavage. In the group of melanocortins is also adrenocorticotropin (ACTH), a derivate of POMC. Another polypeptide (derived with ACTH from POMC) is lipotropin (β and γ), which has central functions in lipolysis, lipid transport, and in steroid genesis. Yet another group of fragments are the endorphins, with proenkephalins A and B, and met-enkephalin. MC3Rs and MC4Rs and POMC neuron activations, further to leptin receptors, have evolved as key components with triggering cardiovascular consequences in MetS (da Silva et al., 2014; Section Melanocortin Receptors and Binding Sites).

### Catecholamine genomic bases and regulation

Stress has long been implicated as a non-metabolic causation in the pathogenesis of MetS (Hjemdahl, 2002), and theoretical accounts of MetS pathophysiology list endocrine factors of stress regulation in reciprocity with factors of cardiovascular regulation, metabolic regulation, and inflammatory regulation (Szczepanska-Sadowska et al., 2010). Short-term stress is mediated in the autonomic nervous system directly by its sympathetic branch, and through transmission by catecholamines, specifically neurotransmitter noradrenaline, under regulation by the catechol-o-methyltransferase (*COMT* 22q11.2) gene (Kopin et al., 1978).

#### Catechol-o-methyltransferase

COMT has been considered repeatedly when investigating the frequent comorbidity of major psychiatric disorders and MetS. The microdeletion syndrome at locus 22q11.2 interrupts *COMT* expression, resulting into a neurodevelopmental schizophrenia phenotype (Napoli et al., 2015), in which increased glycolysis and higher plasma cholesterol and triglyceride concentrations were found. The low-activity allele *COMT* Val158Met polymorphism is related to a subclinical MetS-phenotype involving elevated heart rates, blood pressure, abdominal obesity (Annerbrink et al., 2008). During normal aging, COMT and brain-derived neurotrophic factor (BDNF) showed additive effects on decline in executive functioning in interaction with apolipoprotein E metabolism (Sapkota et al., 2015).

#### Noradrenaline signaling

The SNS is triggered from the amygdala (central and basolateral nuclei) in the presence of stress signals, as human effective connectivity studies have indicated (Lemche et al., 2006). In the brainstem, the main synthesis site in the rostral pons for noradrenaline is the locus coeruleus, besides the adrenal medulla. The projections to the major midbrain and cortical regions are exerted by noradrenergic neurons. We therefore move the focus upon noradrenaline, as the principle neurotransmitter of the SNS. Experimental physiological evidence isolated sympathetic activity and noradrenaline action explaining blood pressure variance (Fossum et al., 2004) during acute stress, thereby potentiating hypertensive action is in MetS (Huggett et al., 2004; Grassi et al., 2007). The adrenoceptor types α_1_ and α_2_ are employed in noradrenaline uptake and signaling, thereby inducing vasoconstriction, and relaxation, respectively, in smooth muscles. Adrenoceptors β_1−3_ are more specifically involved in metabolic processes, binding triggers intracellular concentrations of cAMP as second messenger. Cardiac output, renin, ghrelin (β_1_), and lipolysis, insulin secretion, glycogenolysis (β_2,3_), are mediated by this receptor class, making them relevant for MetS.

#### Adrenoceptor gene loci

In their genomic bases, *ADRB1* (10q25.3) releases heterodimers that influence BMI, body weight regulation, blood pressure, and basic metabolic rate. GWAS have revealed that heart rhythm problems, failure, and blood pressure dysregulation are correlated with this locus (Gao et al., 2014), and its Arg389Gly polymorphism with obesity (Dudchenko et al., 2014). Different polymorphic forms, other variants, and epigenetic modification of the *ADRB2* (5q31-32) gene have been correlated with obesity and T2DM, hyperinsulinaemia, NAFLD, and hyperleptinaemia (Bulatova et al., 2015). These are related to its mediation of hepatic blood flow with glycogenolysis and gluconeogenesis, and insulin secretion from pancreas. *ADRB3* (8p11.23) is mainly expressed in brown and white adipose tissue, and becomes activated in energy expenditure, thermogenesis and lipolysis. Diseases associated with *ADRB3* include obesity based on MC4R deficiency. In addition, the gene is also expressed in the vascular endothelium where it is involved in lipolysis, glucose uptake, cardio-inhibition and relaxation. Hypermethylation of the *ADRB3* gene promoter in blood and visceral tissue is associated with metabolic disturbances (Guay et al., 2014), such as dyslipidaemia. Polymorphisms in the β_3_-adrenoceptor gene were observed with insulin resistance and high lipid profiles related to T2DM and MetS (Burguete-Garcia et al., 2014).

#### Noradrenaline transporter gene

The noradrenaline transporter gene *SLC6A2* (*NET* 16q12.2) is central to noradrenaline homeostasis and presynaptic reuptake. Its SNP *rs*2242446 has been correlated to anxious arousal and PTSD (Pietrzak et al., 2015). *SLC6A2* expression is restricted to noradrenergic neurons that innervate the adrenal medulla. Further links to MetS are in its involvement in cardiovascular regulation, obesity/weight regulation hepatic regulation, which though has not been systematically investigated yet. There is evidence that a SNP (Ala457Pro) in the *NET* gene (*SLC6A2*) may be underlying orthostatic autonomic dysregulation (Tellioglu and Robertson, 2001). An epigenetic mechanism (hypermethylation of CpG islands in the *NET* gene promoter region) that results in reduced expression of noradrenaline has also been described for orthostatic tachycardiac dysregulation, but not been replicated for panic disorder (Bayles et al., 2013).

### Glucocorticoid and 11β-HSD-1 genomic bases and regulation

Two stress pathways, including both the hypothalamic–pituitary–adrenal axis (HPA), and the noradrenergic sympathetic nervous system, have been considered relevant to MetS (Lambert et al., 2010). Long-lasting stress, in specific, is believed being maintained by enduring imbalance of the HPA, through secretion of the corticotropin-releasing hormone (CRH) resulting in hypercortisolism. Short-term sympathetic-noradrenergic action has been linked to state anxiety (Ziegler et al., 2012), whereas trait anxiety and “anxious temperament” (AT; both terms are used interchangeably in neuroscience) is located in hyperactive anxiety midbrain circuits: Anterior hippocampus, amygdala, and ventral striatum have been found to elevate cortisol levels through HPA axis hyperreactivity (Oler et al., 2010; Dinel et al., 2011; Rogers et al., 2013).

#### Corticotropin-releasing hormone gene

Synthesized in the hypothalamic paraventricular nucleus, CRH release is also triggered by TNFα and IL-6 resulting from inflammatory states in order to dampen the immune response, and to adjust endogenous cortisol release controlling the inflammatory response. The expression of *CRH1* (8q13.1) has been related to hypoglycaemia (Nussey et al., 1993), and its activation has been found central to fetal programming of later obesity (Stout et al., 2015). In contrast to SMA activation, cortisol release in response to repeated stress habituates more quickly (Schommer et al., 2003), but leads eventually into immunodeficiency by impairing CD19-promoted B-cell generation (McGregor et al., 2015). Furthermore, a second adipose glucocorticoid system has been described besides to the CNS HPA signaling pathway recently. Here it has been found that also adipose tissue expresses the neuropeptide CRH as part of the inflammatory response (Section Inflammation and Arterial Rigidity), together with in the immune system toll-like receptor-4 (TLR4), the production of inflammatory cytokines IL-6, TNFα and IL-1β, chemokine IL-8, monocyte attractant protein-1 (MCP-1), and of the adipokines adiponectin, resistin, and leptin (Dermitzaki et al., 2014). A second vertebrate corticotropin-releasing hormone gene *CRH2* has recently been discovered but there is still a lacuna in human research (Grone and Maruska, 2015). In humans, the glucocorticoid receptor protein is encoded by *NR3C1* gene, which is located on chromosome 5 (5q31). *NR3C1* mediates the regulatory response to glucocorticoid response elements in the promoters of glucocorticoid responsive genes to activate their transcription, and as a regulator of other transcription factors. It has been found linked to MetS through mechanisms of epigenetic modification by histone methylation in response to early life trauma (Martin-Blanco et al., 2014; Palma-Gudiel et al., 2015) in e.g., personality and eating disorders.

#### Corticotropin receptors and ACTH signaling

There are two subtypes of CRH receptors, both of which express ACTH, when bound by CRH. Corticotropinergic neurons are mainly located in the anterior pituitary, also in amygdala, hippocampus and locus coeruleus. The HPA signaling pathway is mainly dependent on corticotropin-releasing hormone receptor type 1 (CRH_1_) polymorphism on exon 6 of *CRHR1* (locus 17q12-q22 in humans) (Rogers et al., 2013). Within the hippocampus, the CRHR1s are most abundant, but also present in liver tissue. Both *CRHR1* and *CRHR2* genes are strongly expressed in adipose tissue. *CRHR1* (17q21.31) association is frequent in depression, and thus relevant to MetS, since depression is a frequent MetS-comorbidity. The *CRHR2* (7p14.3) gene has been described involved in cardiovascular homeostasis, PTSD, and thus general susceptibility toward stress (Wolf et al., 2013). Their activation product, ACTH, is synthesized in basophile neurons of the anterior pituitary under regulation by CRH from its precursor POMC. ACTH, in turn binds to melanocortin receptors (Section T2DM: Melanocortins and Insulin Resistance).

#### Regulation of cortisol biosynthesis

Cortisol biosynthesis needs as a coenzyme the heme-containing cytochrome P450, secreted after oxidation from cholesterol in adrenal mitochondria in the zona fasciculata to pregnenolon, and also co-regulated by ACTH. The final stage of cortisol synthesis is reached by 11β-monooxygenases (2 isoforms, both *CYP11B1* and *CYP11B2* are on 8q21-q22), adrenal members of the cytochrom-P450 family, and their malfunction will result in missing feedback signaling to ACTH. The gene locus P450 (cytochrome) oxidoreductase (*POR* 7q11.2) encodes the endoplasmic reticulum membrane oxidoreductase, involved in steroidogenesis as well. As the gene product of *POR* is required for the activation of the microsomal P450-enzymes, several hepatic CYP-enzymes are hampered in their activity through mutations pertaining to these functions. Under the aspect of steroidogenesis, are impaired acyl-carbon bond cleaving cytochrome enzymes CYP17A1, CYP21A2, and CYP19A1. Respective mutations in their gene loci were observed relevant to the regulation of lipid traits, in specific hypercholesterolaemia, and blood pressure (Lu et al., 2015).

#### Cortisone-cortisol interconversion

11β-dehydroxysteroid dehydrogenase (HSD-11β) is a catalytic enzyme and a membrane protein of the endoplasmic reticulum converting free cortisol to inactive cortisone (and in type 1 isozyme, also vice versa through 11-oxidoreductase activity), in two isoforms 1 and 2, and eight known structures. As mentioned, isoform 1 also performs the reduction of cortisone to active cortisol in CNS, liver and adipose tissue, and, as has been suspected, thereby amplifying cortisol action (Seckl and Walker, 2001).

Isoform 2 oxidizes free cortisol to cortisone, a process seen in placenta, testes, lungs, or kidneys. For this reason 11β-HSD-2 has been implicated in neurodevelopmental susceptibility for the programming of diathesis toward chronic stress (Sousa, 2016). By this conversion it is prevented that the abundant ligand cortisol binds to the mineralcorticoid receptor (MR) in addition to the glucocorticoid receptor, thus securing only aldosterone being able binding to MR. The HSD-11β conversion activity is mainly a process in adipose tissue, with its distinct CRH-system described above, but exerting feedback effects upon the CNS HPA axis. It has therefore been proposed counteracting HSD-11β in adipose tissue as a treatment for MetS by both pharmacological and psychological interventions on central HPA and peripheral cortisol systems (Anagnostis et al., 2009). Green tea has shown potency to prevent hepatic cortisol activation by type 1 isozyme HSD-11β (Hintzpeter et al., 2014). Because of its amplificatory action of active glucocorticoids, isoform 1 HSD-11β, has been assumed be a pathogenic factor in MetS, T2DM, and age-related cognitive decline (Seckl and Walker, 2001).

#### HSD-11β gene loci

The protein encoded by the *HSD11B1* (1q32-q41) gene is a microsomal enzyme that catalyses the conversion of the stress hormone cortisol to the inactive metabolite cortisone and reverse (see above). Too much cortisol can lead to central obesity, and several variations (*rs*10082248, *rs*2298930, and *rs*4545339) in this gene have been associated with obesity and insulin resistance in children (Ruan et al., 2014). Cortisol interconversion is mainly relevant at the visceral tissue level (Kilgour et al., 2015). There is initial evidence that *HSD11B1* expression predicts insulin resistance (Koska et al., 2006; Gyllenhammer et al., 2014), and also a linear relationship between BMI and 11β-HSD1 has been observed when pooling across samples (Wake and Walker, 2006). Initial evidence also suggests that *HSD11B1* expression is likely to be regulated in a tissue-specific manner (Wake and Walker, 2006; Wake et al., 2006), i.e., present in different levels in adipose tissue and mainly liver (Stimson and Walker, 2013). The regulation of both 11β-HSD isoform-genes is dependent on NF-κB (Lee et al., 2013) in adipose tissue. Both isoforms have been found relevant to childhood obesity (Ruan et al., 2014), the antecedent of MetS. In interaction with adiponectin, *HSD11B1* determines the metabolic rate already *in utero* and postnatal development (Muramatsu-Kato et al., 2014), in a form of ontogenetic programming. *HSD11B2* (16q22) protects cells from the growth-inhibiting and/or pro-apoptotic effects of cortisol, particularly during embryonic development. Mutations in this latter locus cause the syndrome of apparent mineralocorticoid excess and hypertension. Polymorphisms can regulate maternal cortisol levels *in utero* and regulate postnatal weight gain (Rogers et al., 2014). Replicated in association studies were polymorphisms for salt sensitivity, RAAS, and essential hypertension. Negative findings were yielded for *HSD11B2* SNPs with adolescent obesity (Ruan et al., 2014); similarly, gene regulation studies identified only microRNA relevant to renal functioning (Rezaei et al., 2014). However, the polymorphic CA-repeat polymorphism in the first intron of *HSD11B2* was significantly related to insulin insensitivity (Mune et al., 2013).

### Adiponectin and genomic bases

Adiponectin is a regulatory peptide hormone secreted by adipocytes (but also by myocytes)—less when emptied, more when filled with lipids—relevant to glucose flux and lipid catabolism. A low adiponectin level in obese persons attenuates insulin action in tissue by induction of insensitivity via modification of its uptake by adipocytes (Ali et al., 2013). Such a condition is therefore conducive to insulin resistance and T2DM, but at the same time protective for endothelial tissue (Fortuño et al., 2003). High adiponectin levels, in contrast, are known for insulin sensitization and anti-inflammatory reflexes, whereas low levels have, however, been shown to be an independent risk factor for Alzheimer's and other dementias (van Himbergen et al., 2012). In addition, inverse relations between adiponectin and cortisol secretion have been described, whilst plasma cortisol being the best predictor of insulin resistance (Lehrke et al., 2008), however, the precise physiological mechanism of this antagonism remains unclear.

#### Release of adiponectin

The adiponectin gene is known being expressed only in adipose tissue: *ADIPOQ* (3q27) with five exons expresses collagen-like proteins and co-factors. It has been associated with rapid excessive weight gain, obesity, low-frequency heart rate variability and cardiac mortality (Alehagen et al., 2015; Riestra et al., 2015), and negatively with risk for T2DM and CVD incidences (Lindberg et al., 2015). Complementary functions with leptin (see Section Leptin and Ghrelin Receptors and Binding Sites) were described for vascular injury (Fortuño et al., 2003). Low level of adiponectin is demonstrated being a singular independent risk factor for developing MetS (Díez and Iglesias, 2003; Renaldi et al., 2009). Direct interactions of adiponectin have presently been documented mainly with the other adipokines (Raucci et al., 2013; Sitticharoon et al., 2014), but first of all leptin (Section Leptin and Ghrelin Receptors and Binding Sites).

#### Adiponectin receptors

Adponectin binds to three receptor types receptors (AdipoR1, AdipoR2, T-cadherin CHD13), which activate the hepatic and pancreatic enzyme 5′ AMP-activated protein kinase, p38-MAPK, a mitogen-activated protein kinase sensitive to stress, to pro-inflammatory cytokines, hepatic PPARα related to triglycerides, and to NF-κB transcription factor related to stress and inflammatory response (Thundyil et al., 2012). It has recently been described that adiponectin and its receptors modulate analgesic effects in the central nervous system further to its anti-inflammatory properties (Iannitti et al., 2015). The adiponectin receptors are ubiquitous in the cerebrum, but more densely expressed in pituitary, hypothalamus, brainstem, cortical neurons and endothelial cells. Recent findings suggest that adiponectin has neuroprotective properties and counteracts cerebral apoptosis (Song J. et al., 2015). Adiponectin receptors and binding (Tilg and Moschen, 2006) differ between receptor types: whereas *ADIPOR1* is expressed ubiquitously in muscular tissue, *ADIPOR2* is restricted to hepatic tissue. The binding is dependent on molecular-weight homomultimer forms of the specific molecule. Both receptors are known to mediate fatty-acid oxidation and glucose uptake (Yamauchi et al., 2003). *ADIPOR1* (1q32.1) was associated with insulin resistance, T2DM, and CAD, specified to different susceptibility SNPs (Jin et al., 2014). *ADIPOR2* (12p13.31) exhibited correlations with CAD, stroke, and T2DM (Yuan and Teng, 2014). The *CHD13* (16q23.3) protein regulates axon growth during neural differentiation and vascular endothelial cells from apoptosis due to oxidative stress, and is associated with resistance against atherosclerosis. The interaction of its SNPs and methylation has not yet been fully understood, but relations were found to diastolic blood pressure and HDL levels (Putku et al., 2015). Besides adiponectin, at least 15 other substances are subsumed to the category adipokines (Raucci et al., 2013), but outside the scope of this treatise.

## Brain regions of MetS neuroendocrine signaling systems

### Leptin and ghrelin receptors and binding sites

White adipose tissue regulates sympathetic output through release of peptides such as the proteohormone leptin, which have the ability to cross blood brain barrier and bind to receptors in higher ANS regions (Thorp and Schlaich, 2015). Leptin is a peptide hormone produced by adipose tissue in proportion to fat mass. The physiological function of leptin release is (a) to promote food intake, and (b) to increase energy expenditure (Barnes and McDougal, 2014). In obesity, leptin is assumed to drive sympathetic activity and to contribute to hypertension (Hall et al., 2010). Its central function is the maintenance of homeostasis in fat depot metabolism. Leptin functioning is, however, also dependent on the brain melanocortin system (da Silva et al., 2014; Bassi et al., 2015b). Leptin inhibits hunger sensations, and therefore, a lack of satiation feelings is induced by deficient cerebral leptin feedback signaling (Moreira et al., 2015). Such anorexigenic signals are mediated by leptin and insulin via hypothalamic POMC neurons activating MC4Rs. Leptin induced obesity states are linked to ROS increase in oxidative stress (Section Inflammation and Arterial Rigidity). A counterintuitive effect of leptin is present in its reinforcement of sympathetic action on the adrenocortical system, but an important link to hypertension. This accounts for its interrelations with the cardiovascular system, the sympathetic ANS branch, metabolism, and obesity, and with chemoreceptors in the carotid bodies (Bassi et al., 2015a; Zeng et al., 2015). Leptin depletion in both animals and humans results in a quasi-MetS condition, but lacking essential hypertension and sympathetic hyperexcitability. It is concluded from these depletion states in leptin levels that insulin resistance and hyperinsulinaemia, hyperglycaemia, dyslipidaemia and visceral adiposity could also be related to leptin action (Bassi et al., 2015a). As mentioned, this requires the POMC and MC4R neurons to be activated: Mice with MC4R deficiency are (a) unresponsive to leptin, and (b) also develop artificial MetS symptoms analogous to those in leptin depletion, despite high leptin levels (Kooijman et al., 2014). Adiponectin and leptin levels are typically positively associated (Singh et al., 2016), but this correlation is absent in obese persons: Adipose tissue from obese subjects has impaired leptin signaling, which probably prevents increases in adiponectin levels in obese.

#### Leptin biosynthesis

The protein leptin is a synthesis product of white adipocytes, expressed by the *LEP* (7q31.3) gene, active in haematopoiesis, angiogenesis, healing processes, immune, and inflammatory responses. To the degree that lipolysis is exerted in fat depots, plasma concentration of leptin diminishes, thus raising appetite. Besides in adipocytes, leptin is also produced by neurons in hypothalamus and pituitary (Morash et al., 1999). Leptin deficiency caused by null mutations (Mark, 2013) induces components of MetS such as hyperinsulinaemia and hyperlipidaemia, but sympathetic hypoactivation. In contrast, chronic leptin infusion leads to elevated blood pressure, under engagement of adrenoceptor activity. It is likely that leptin upregulation is a biomarker for chronic obesity-related inflammation from childhood onward (Reyes et al., 2015). Association of the *LEP* gene with severe obesity and T2DM have been reported, also case studies in *LEP* gene malfunction leading to binding-inactive leptin with infantile hyperphagia (Wabitsch et al., 2015).

#### Leptin receptors

The leptin receptor gene *LEPR* (also *LPR, OB-R*, or *CD295* 1p31.3) is crucial for the satiation signaling pathway (Clément et al., 1998; Rolland et al., 1998), and null mutations lead to early onset childhood obesity, disturbance of the somatotropic axis and loss of puberty. Leptin receptors were identified in distinct neuron populations of the arcuate and paraventricular nuclei of the hypothalamus. The first group produce the AgRP and neuropeptide Y, inhibited by leptin. The second group produce α-MSH, and are activated by leptin, which is alike inhibitive of appetite. Leptin receptors, which are in regulation of blood pressure, have also been discovered in the rostroventral lateral medulla (RVLM). Recent experiments demonstrate that specifically adrenal sympathetic activity was steered by RVLM through leptinergic neuronal projections into the kidneys (Barnes and McDougal, 2014), further to regulating general mean arterial pressure. It is possible that, in obesity and T2DM, a deficiency in leptin uptake and binding is present due to high levels of triglycerides then suppressing leptin action (Oswal and Yeo, 2010). Of the leptin-receptor polymorphisms (Gln223Arg, Lys656Asn, and Lys109Arg), the presence of the Arg223 homozygous or the Asn656 allele was associated with elevated plasma leptin, BMI, waist circumference, and waist-to-hip ratio, but less catecholamine presence and dampened sympathetic activity (Masuo et al., 2008). Other mutations in *LEPR* have been identified causative for childhood obesity (Huvenne et al., 2015). Loci coupled to *LEPR* are *LEPROT* (1p31.3) and *LEPROTL1* (8p12), modifying leptin receptor signaling and triggering expression of hepatic growth hormone receptors (Touvier et al., 2009). Copy number variations in these loci are as well associated with obesity, T2DM, and metabolic rates (Couturier et al., 2007; Jeon et al., 2010).

#### Ghrelin physiological functions and binding

Ghrelin is growth hormone release inducing, as it activates the growth hormone secretagogue receptor in the hypothalamus, which results in the secretion of growth hormone (somatotropin). Further to leptin, ghrelin is involved with hunger-satiation-regulation in the hypothalamic nucleus arcuatus. Ghrelin is the “hunger hormone” and in many ways antagonistic to leptin action. Orexigenic signals are mediated by ghrelin via NPY receptors, inhibiting MC4Rs. It is released from ghrelinergic cells in the gastric mucosa, ε-cells of the pancreas, and the hypothalamus and anterior pituitary. The peptide hormone ghrelin is derived from posttranslational modification by cleavage from its precursor obestatin (Zhang et al., 2005), a peptide with opposing effects. Hunger states or sleep deprivation increase ghrelin levels, which would decline postprandially. Ghrelin stimulates NPY and agouti-related hormone (AgRP) action in the arcuate nucleus.

The ghrelin/obestatin gene *GHRL* (3p26-p25) produces these two products. Ghrelin is involved in energy homeostasis, and hereby regulates pancreatic glucose-stimulated insulin secretion. Obestatin also has multiple metabolic functions, but is regulative for adipocyte and glucose metabolisms. Low ghrelin level confers CVD and other risks, whereas high plasma ghrelin levels have vasoproctective effects (Laurila et al., 2014). The ghrelin receptor (*GHSR* 3q26.31 producing two transcripts, of which only type 1a is a receptor for ligand ghrelin) is found on the same cells in the brain as the leptin receptor. It activates hedonic dopaminergic neurons in the mesolimbic cholinergic–dopaminergic pathway that processes reward-related activation between ventral tegmental area and nucleus accumbens; possibly intragastric metabolic nutrient-sensing signals toward dorsal striatum (Stuber and Wise, 2016). It is primarily involved in the modulation of glucose and lipid metabolism, digestion, neuroprotection, and regulation of immune functions. Ghrelin receptors have high density in the hypothalamus and pituitary, and on the vagus nerve (on both afferent cell bodies and afferent nerve endings) and throughout the gastrointestinal tract. Meta-analytic analyses on the polymorphisms in *GHSR* indicated that these are in regulation of blood glucose (Pabalan et al., 2014). The gene for the obestatin receptor *GPR39* (2q21-q22) may have functions in repair and wound healing, and is upregulated by antidepressants (Mlyniec et al., 2015).

### Melanocortin receptors and binding sites

Two types of melanocortin receptors have increasingly been placed central to MetS pathophysiological understanding, but in specific the MC4R (Section T2DM: Melanocortins and Insulin Resistance). The reason for that is due to their capability to bind also other ligands: POMC and MC4R neurons are necessary for leptin signaling, on the one hand, and on the other hand suppresses AgRP as ligand in MC4Rs their respective activation. The brain melanocortin system is embedded in leptin, ghrelin, and agouti-related energy and feeding homeostatic systems, as well as glutamatergic eating-reward behavior circuitry between lateral hypothalamic area, amygdala and VTA (Stuber and Wise, 2016). Leptin binds to leptin receptors (LEPRs) on AgRP-secreting neurons and POMC-secreting neurons in the arcuate nucleus of the hypothalamus. Leptin binding suppresses AgRP synthesis and triggers the production of POMC, which is the precursor for α-, β-, and γ-MSH: AgRP is the main antagonist of MC4R (Fani et al., 2014). Accordingly were cardiovascular and metabolic actions of leptin abolished in obese and non-obese MC4R deficient mice (Tallam et al., 2006). Stimulation of the MC4R causes a decrease in appetite and an increase in metabolism of fat and lean body mass: According to current account are ARC POMC and PVNH MC4R, and LPBN neuron populations responsible for nutrient chemosensation and relay of satiety evaluations to visceroceptive forebrain regions (Krashes et al., 2016). Functionality of MR4R is also necessary to induce blood pressure changes and metabolic alterations (Tallam et al., 2005; da Silva et al., 2006). Morbid obesity is associated with silencing of MC4R activation. In contrast to AgRP-related blockade of MC4R activation, stimulates α-MSH binding MC4R activation toward steering food intake via BDNF signaling to higher cerebral centers (Walley et al., 2009). Input to POMC neurons to trigger the brain melanocortin system requires (a) afferent vagal input of hunger signals via its dorsal motor root nucleus and NTS (Krashes et al., 2016), (b) leptin adipostatic signals crossing the blood-brain barrier, and (c) gut-released peptides cholecystokinin, ghrelin and PYY uptake (Cone, 2005; Ellacott et al., 2006). Its telencephalic network has not yet been exactly mapped, but is quite likely to comprise dysgranular cortices in visceroceptive insula, operculum, and in the caudolateral orbital frontal regions (Batterham et al., 2007).

#### Hypothalamic and brainstem nuclei

Distributions of MC3Rs and MC4Rs are widespread, and in relation to energy homeostasis, but specifically MC4Rs in paraventricular hypothalamic nucleus (PVNH) and amygdala are related to food intake behaviors (Balthasar, 2006). Basically, there are three main melanocortin circuits with (a) hypothalamic POMC neurons in the arcuate nucleus, in relation with the agouti-related neuropeptide and neuropeptide Y expressing neuron populations, (b) brainstem POMC neuron population in the commissural nucleus of the tractus solitarius, and (c) the telencephalic target system of MC3R and MC4Rs. Driving of sympathetic output is exerted mainly by allostatic excitatory neurons in the subfornical organ (SFO) (Wei et al., 2013; Young et al., 2013; Oka et al., 2015), and by the caudal vasomotor brainstem through leptin receptors. Leptin receptors are there expressed by adrenergic/noradrenergic C1/A1 cells, which overlap in the rostral ventrolateral medulla (RVLM) with melanocortin neurons (Grassi et al., 1998).

#### MSH signaling

α-, β-, and γ-MSH bind to the MC4R receptor, a G-coupled transmembrane receptor, and this activates the brain melatonin system (da Silva et al., 2014). MC3R and MC4R are widespread over the brain, but only MR4R blockade results in hyperphagia causing obesity, and MC4R binding triggers energy expenditure while diminishing appetite (Tallam et al., 2005). Other proteins activating the MC4R are ACTH and POMC. However, *MC2R* (18p11.2) is also target of ACTH, and related to familial glucocorticoid deficiency, and/or blunted cortisol responding. *MC3R* (20q13.2-q13.3) increased fat mass despite decreased food intake, energy homeostasis, and also binds MSH and ACTH. Defects in *MC4R* (18q22) lead into an ontogenetic obesity phenotype with autosomal dominant obesity (Farooqi et al., 2003a,b). In childhood obesity, significant SNPs were its Val95Ile, Val166Ile, and Val179Ala mutations, which were primarily related to plasma lipid levels (Song et al., 2015). With regards to MetS, *MC4R* is the largest known single risk factor for combined obesity and T2DM manifestation, as replicated by several GWAS studies (Chambers et al., 2008; Loos et al., 2008; Thorleifsson et al., 2009; Willer et al., 2009). However, until 2014 more than 80 distinct mutations in its locus have been described (Fani et al., 2014). This finding is distinct from those in non-syndromal obesity (Walley et al., 2009), where *FTO* and the ghrelin receptor *GHSR* exhibited the largest odds ratios.

#### Expression of melanocortin receptors

Brain regions with highest levels of MC3R (Begriche et al., 2011) are the midbrain structures. MC3Rs are densely expressed in hypothalamic and limbic regions of the brain and in peripheral tissues. The ventromedial hypothalamus (VMH), a critical node in the neural circuits regulating feeding-related behaviors and metabolic homeostasis, exhibits dense MC3R expression. MC3R has been related to increased fat mass, and accelerated diet-induced obesity (DIO). MC3R causes in accordance with biorhythmic cycling hyperinsulinaemia, glucose intolerance, increased expression of lipogenic genes, and increased ketogenesis. Rhythmic expression of MC3R transcription factors was found regulating liver clock activity (Sutton et al., 2010). The brain regions with highest levels of MC4R (Rossi et al., 2011) are brainstem neurons including those in the dorsal motor nucleus of the vagus, paraventricular nucleus of the hypothalamus (PVNH), the amygdala, nucleus tractus solitarius, intermediolateral medulla (IML) (Krashes et al., 2016). Brain regions critically subserving the hunger-satiation regulation were described to consist in MC4R neuron populations in the PVNH toward the lateral parabrachial nucleus (LPBN) pathway (Garfield et al., 2015). A distinct interaction between MC4Rs in the PVNH and amygdala has been found sufficient to control food intake behaviors (Balthasar, 2006). The central MC4R pathway is also crucial for control of cholesterol metabolism by the liver, specifically in determining the HDL/LDL ratio (Perez-Tilve et al., 2010), a process decisive for the MetS constellation and CVD, if derailed. Regarding energy expenditure, a crucial role has been assigned to the circumventricular structure organum vasculosum laminae terminalis lacking the blood-brain barrier (Oka et al., 2015). This structure adjacent to cerebral ventricles receives input by IL-1β signaling and triggers TNFα synthesis. It has anatomical connections with the hypothalamic nucleus praeopticus (Oka et al., 2015), which relays output to the periaquaeductal gray (PAG) and Raphe nuclei, all related to thermogenesis (Cone, 2005).

### Neuropeptide Y and agouti-related hormone receptors and binding sites

The likely neurophysiological linkages between sympathetic stress reactivity, the glucocorticoid system, and the melanocortin system are the neuropeptide Y (NPY) and the AgRP systems. These hormones are the major orexigenic signals in the hypothalamus. Both sympathetic nerves and immune cells are capable to produce NPY, which has a protective buffering function against the immune challenges exerted by low-level inflammation (Farzi et al., 2015), and contributes to resilience against environmental, inflammatory, and oxidative stress.

#### Physiological function of neuropeptide Y

The prevalence of NPY is abundant in the cerebrum, but major sites of action are the hippocampal formation, the amygdala and septum (Thorsell, 2010), with highest prevalence rates in cortices, limbic system, and hypothalamus. Its effects are to increase cortical excitability, while alerting with cardiovascular responsiveness, to raise intracellular calcium levels, and thus activating potassium channels. These latter processes exert vasoconstriction, while at the same time lowering blood pressure. However, a somatotropic function of NPY is in promoting growth of adipose tissue (Kuo et al., 2007). In addition, it has been shown that NPY modulates neurogenesis. Interestingly, these latter effects are similar to those elicited by neurotrophins (Angelucci et al., 2014). In sum, the activity effects of NPY are in alertness, anxiolysis, analgesia, fat deposition, and orexigenesis. Obesity states are characterized by increases in NPY mRNA and NPY release (Dryden et al., 1995).

Release of NPY in the PVNH triggers CRH, as there is also a negative feedback loop of CRH on NPY synthesis and release. In this reciprocal circuit mechanism, NPY action is a direct stimulation of synthesis and release of CRH and vice versa. This reciprocal interaction of CRH-NPY with a tension-vs-anxiolysis effect was concluded to be an internuclear interaction mechanism within the amygdala, specifically the basolateral amygdalar nucleus (BLA) (Thorsell, 2010), which is known to trigger anxiety responses, and perhaps the central amygdaloid nucleus (CeA). Furthermore, NPY concentrations were significantly higher in the medial praeoptic area (MPO), paraventricular (PVNH), ventromedial (VMN), and dorsomedial (DMN) nuclei of the hypothalamus. It is therefore likely that NPY synthesis and release participates in the emergence of T2DM. High levels of glucocorticosteroids stimulate gluconeogenesis, which increases blood glucose, triggers release of insulin. Insulin action is to reuptake and store glucose as glycogen, while insulin resistance finally results in T2DM.

#### Biosynthesis and gene

In general, neuropeptide Y is essential in controlling cortical electrophysiological excitation, and thus in suppressing epileptic processes. Its intranasal application reduces PTSD symptoms pharmacologically. The *NPY* gene (7p15.1) has just one documented SNP, the Leu7Pro7 mutation, associated with elevated cholesterol, so conferring specific risks for MetS and CVD. In that polymorphism, specifically, abdominal fat deposition correlated with the *rs*16147 C and T alleles (Lin et al., 2015). The *NPY* is expressed by SNS neurons as an ubiquitous cerebral neurotransmitter that is not at all restricted to hypothalamus. However, neuropeptide Y (NPY) is in particular synthesized in arcuate (ARC) neurons, which then project principally to the paraventricular nucleus (PVNH). Axon terminals in PVNH also contained the highest levels of NPY immunoreactivity, together with the arcuate nucleus. NPY directly injected into the PVNH caused hyperphagia, reduced energy expenditure, and eventually produced obesity.

#### Receptor subtypes and their genomic associations

There are up to six subtypes of NPY receptors currently known, but only four of which are known to mediate neuroendocrine responses: NPY1R, NPY2R, NPY4R, and NPY5R (Thorsell, 2010), regulation of food intake is mediated only by NPY1R and NPY5R receptors. The *NPY1R* (4q32.2) exerts anxiolytic activation (Olesen et al., 2012b), triggers mobilization of intracellular calcium and inhibition of adenylate cyclase activity. Variants in *NPY1R* were found related to heritable autonomic traits in circulation, baroreflex functioning, and pressor response to environmental stress (Wang et al., 2009). Expression of *NPY1R* in adipose tissue was observed relating to the MetS components weight and insulin resistance (Sitticharoon et al., 2013). *NPY2R* (4q31) participates in modification of cardiometabolic traits (Wei et al., 2013), with its 21 known SNPs. Haplotypes of the proximal promoter variants G-1606A, C-599T, and A-224G disrupted predicted dependent genes, influenced the transcription of the interferon regulation factor related to IL-6, and the hepatocyte nuclear factor. *NPY4R* (10q11.2) gene products are the targets of the pancreatic polypeptide, which, in the adrenal medulla, locally enhances the secretion of catecholamines (Cavadas et al., 2001). *NPY5R* (4q32.2) activation results in behavioral hyperactivity (Olesen et al., 2012a), in epilepsy suppression (Gøtzsche et al., 2012), and in regulating food intake, while defects in this gene being associated with eating disorders and BMI (Li et al., 2014).

Rodent studies have identified the *NPY2R* activation as critical for an animal model of MetS. In this context it was possible to show that stress consisting of environmental or social stressors trigger NPY release from sympathetic nerves, which in turn sensitizes its NPY2Rs for glucocorticoid-dependent action in the abdominal fat depot (Kuo et al., 2007). Visceral fat accumulation is thus a consequence of *NPY2R* activation, which incites proliferation of adipocytes, triggers angiogenesis in adipose tissue, and promotes migration of macrophages into adipose tissue (Ali et al., 2013). In this accumulation of visceral fat were glucocorticoids described as central in stress-mediated exacerbation of DIO. Respective results suggested that glucocorticoids become modulators of NPY-NPY2R signaling. In the presence of high-calorie nutrition, plasma corticosterone levels, a precursor of aldosterone, raise, and in such a constellation, sympathetic excitation increases the conversion of the inactive steroid precursor to active cortisol by upregulating 11β-HSD-1 activity, specifically in the abdominal fat (Kuo et al., 2007).

The catecholamine hormone adrenaline has been shown to upregulate *NPY* and *NPY2R* expression *in utero* (Han et al., 2012) in dependence of the glucocorticoid system. Prenatal stress accelerates adipogenic programming of embryonic stem cells with adrenaline during their adipogenic differentiation.

#### Agouti-related neuropeptide

Agouti-related neuropeptide (AgRP) is only synthesized in neurons, which also contain NPY (Bäckberg et al., 2004; Krashes et al., 2016), mainly in the arcuate nucleus, but also PVNH, of the hypothalamus, and which also contain ghrelin receptors. Its main expression sites are, besides the hypothalamus, the subthalamic nucleus, and the adrenal medulla. Starvation sensations have been shown to be dependent on the output of AgRP populations in ARC toward PVNH pathway (Atasoy et al., 2012), which are abrogated by food stimuli in interplay of AgRP and SFO neurons (Betley et al., 2015). The AgRP neuron population, in turn, is inhibited by MC4R neurons in the PVNH, mediated by a circuit between PVNH and lateral zone LPNH (Garfield et al., 2015). AgRP also stimulates the HPA to release ACTH, cortisol and so counteracts pro-inflammatory cytokine IL-1β (Xiao et al., 2003). The expression of *AGRP* (16q22) in the adrenal gland blocks α-MSH-induced secretion of corticosterone (Dhillo et al., 2003). Acute stress response, in turn, downregulates *AGRP* expression as measured by its mRNA. High plasma AgRP levels are a further correlate of obesity. The main physiological function of AgRP is in the hypothalamic control of food intake by means of intracellular calcium levels. AgRP and NPY are secreted to increase appetite, triggered by pro-inflammatory cytokines to decrease metabolism and energy expenditure. It also enhances the ACTH response toward IL-1β, suggesting it may play a role in the modulation of neuroendocrine response against inflammation. It also serves as endogenous antagonist of the MC3Rs and MC4Rs, which blocks binding of α-MSH presumably through competitive ligand binding. An enduring AgRP-induced blockade of MC4R leads to hyperphagia and obesity. Polymorphisms in the *AGRP* (16q22) locus have been linked with anorexia nervosa (Vink et al., 2001), furthermore in cognitive functioning there, and to late onset obesity in its *rs*11575892 T allele (Kalnina et al., 2009). The polymorphism Ala67Thr is, however, protective against this obesity risk (Sözen et al., 2007).

## Findings on epigenetic modification and gene regulation in MetS

The past decade has seen the conduction of studies elucidating the posttranscriptional and posttranslational activity of genes presumably involved in MetS, as specified in the preceding sections. Specific new evidence accumulated in the context of studies in “programming” of these genes in effect of interaction with environmental processes, early or later in life.

### Epigenetic modifications

The risk gene loci for the development of MetS identified in the previous parts were entered to a meta-analytic evaluation. Table 1 lists the findings describing epigenetic processes histone methylation, lysine acetylation, serine phosphorylation, and ubiquitination. As can be seen, most results are currently pertaining to POMC neurons in the hypothalamus. In summary, it can be said that several studies support the presence of early life stress (ELS) and DIO programming in this convergence region. Furthermore, the results suggest tissue-specific modification of risk gene expression in the midbrain, liver, and adipose tissue. With regards to neuroendocrine systems, epigenetic modifications were found in cortisol-related (here *HSD11B1* expression), melanocortin, leptin, *NPY*, and adiponectin genes. With respect to adiposity genes, epigenetic modifications were documented for fat mass gene cluster *APOA1/C3/A4/A5*, and the lipolysis gene *LIPE*. With regard to inflammatory, immune and subcellular metabolism, *PPARG, NKBF1, TNFA, TCF7C2*, and those genes expressing members of the cytochrome P450 family that are involved in steroidogenesis and in relation to hepatic lipoproteins. These results on MetS are more consistent than those previously published on obesity (van Dijk et al., 2015). Studies directly pertaining to core HPA axis are very few up to present, with < 10 entries in PubMed: In this context, existing studies addressed the *NR3C1* and *CRH1* loci, with one study (Bockmühl et al., 2015) related to MetS. In sum, there is a lacuna of research in this area.

**Table 1:** **Risk genes and findings on epigenetic modification in the metabolic syndrome**.

**Gene name**	**Gene locus**	**Type of modification**	**References**	**Text section**
*ADIPOQ*	3q27	Subcutaneous and visceral adipose tissue CpG islands associated with *LEP* and *ADIPOQ* gene expression	Houde et al., 2014	3.5
*ADIPOR1*	1q32.1	Methylation changes after exercise in T2DM relatives	Nitert et al., 2012	3.5
*ADIPOR2*	12p13.31	Negative		3.5
*ADRB1*	10q25.3	Negative		3.3
*ADRB2*	5q31-q32	*ADRB2, ADRB3* expression significantly lower in visceral adipose tissue	Kurylowicz et al., 2015	3.3
*ADRB3*	8p11.23	*ADRB2, ADRB3* significantly lower in visceral adipose tissue. *ADRB3* DNA methylation levels significantly associated with LDL and higher BP	Guay et al., 2014; Kurylowicz et al., 2015	3.3
*AGRP*	16q22	Reduction of AgRP and α-MSH-fibers in the paraventricular nucleus with protein restriction *in utero*	Coupé et al., 2010	4.3
*APOA1*	11q23-q24	DNA methylation profiles in *APOA1/C3/A4/A5* gene cluster interrelated	Guardiola et al., 2014	3.1
*APOA4*	11q23	DNA methylation profiles in *APOA1/C3/A4/A5* gene cluster interrelated	Guardiola et al., 2014	3.1
*APOA5*	11q23	DNA methylation profiles in *APOA1/C3/A4/A5* gene cluster interrelated	Guardiola et al., 2014	3.1
*APOB*	2p24-p23	Promoter hypomethylation of *APOB* for pro-inflammatory M1-macrophage, and hypermethylation of anti-inflammatory, pro-angiogenic M2-macrophage genes in hyperlipidaemia and T2DM	Babu et al., 2015	3.1
*APOC3*	11q23.3	DNA methylation profiles in *APOA1/C3/A4/A5* gene cluster interrelated	Guardiola et al., 2014	3.1
*APOE*	19q13.2	*APOE*(^−∕−^) mice: mechanosensitive genes suppressed by disturbed flow by hypermethylation in their promoter region	Dunn et al., 2015	3.1
*CHD13*	16q23.3	Negative		3.5
*COMT*	22q11.21	Negative		3.3
*CRH1*	8q13	Negative		3.4
*CRHR1*	17q21.31	Negative		3.4
*CRHR2*	7p14.3	Negative		3.4
*CYP11B2*	8q21-q22	Negative		3.1
*CYP11B2*	8q21-q22	Negative		3.1
*CYP17A1*	10q24.3	Genes involved in liver metabolism *CYP3A5* and steroidogenesis *CYP17A1* and *CYP19A1* affected by methylation/deacetylation	Dannenberg and Edenberg, 2006	3.1
*CYP19A1*	15q21.1	Genes involved in liver metabolism *CYP3A5* and steroidogenesis *CYP17A1* and *CYP19A1* affected by methylation/deacetylation	Dannenberg and Edenberg, 2006	3.1
*CYP21A2*	6p21.3	Negative		3.1
*CYP2C19*	10q24	Negative		3.1
*CYP3A5*	7q21.1	Genes involved in liver metabolism *CYP3A5* and steroidogenesis *CYP17A1* and *CYP19A1* affected by methylation/deacetylation	Dannenberg and Edenberg, 2006	3.1
*CYP4A11*	1p33	Negative		3.1
*FTO*	16q12.2	*FTO* both DNA methylation and expression correlated with BMI. DNA hypomethylation of CpG site located in intronic region within *FTO* is associated with impaired glucose metabolism and T2DM. CpG sites of *TCF7L2, FTO* with differential DNA methylation in T2DM islets	Bell et al., 2010; Almén et al., 2014; Dayeh et al., 2014; Rönn et al., 2015; Toperoff et al., 2015	3.1
*GHRL*	3p26-p25	Negative		4.1
*GHSR*	3q26.31	Negative		4.2
*HSD11B1*	1q32-q41	*HSD11B1* promoter hypomethylation in liver varied according to diet in ontogeny. Hypomethylation in *HSD11B1*. P1 promoter in association with increased 11β-HSD-1 oxidoreductase activity in T2DM	Inder et al., 2012; Takaya et al., 2013	3.4
*HSD11B2*	16q22	Relation of CpG islands with fetal growth and greater birth weight, with maternal stress, indicating that greater stress is related to gene activity. Similarly, prenatal maternal strain associated with less methylation to provide greater postnatal gene expression, with sex-specific transcriptional regulation. Hypermethylation in *HSD11B2* promoter is related to hypertension and atherosclerosis	Friso et al., 2008; Appleton et al., 2013; Green B. B. et al., 2015;	3.4
*IL1B*	2q14	Negative		2.3
*IL6*	7p21	Negative		2.1
*IL6R*	1q21	Negative		2.1
*LEP*	7q31.3	Subcutaneous and visceral adipose tissue CpGs associated with *LEP* and *ADIPOQ* gene expression in adipose tissues. Maternal fasting glucose associated with *LEP* CpG and hypomethylation determinative of umbilical cord blood leptin levels	Houde et al., 2014; Allard et al., 2015	4.1
*LEPR*	1p31	Negative		4.1
*LIPE*	19q13.2	Adipose tissue DNA methylation in *LIPE* gene associated with adiposity subtypes	Agha et al., 2015	3.1
*MC3R*	20q13.2-3	Negative		4.2
*MC4R*	18q22	Negative		4.2
*NFKB1*	4q24	Nutrient supplementation altered *NFKB1* expression in liver cells during ontogeny. Maternal increased methylation at *NFKB1* CpGs determinative for offspring cord blood DNA	Morales et al., 2014; Osorio et al., 2014	2.3
*NPY*	7p15.1	Altered methylation of specific CpG *NPY* in hypothalamus according to diet in ontogeny. Selective downregulation after high-fat diet in ontogeny in hypothalamus	Mahmood et al., 2013; Cifani et al., 2015	4.3
*NPY2R*	4q31	Negative		4.3
*NR3C1*	5q31.3	Early life stress programs the expression *NR3C1* by site-specific hypermethylation at the CpG island shore in CRH producing hypothalamic neurons	Bockmühl et al., 2015	3.4
*POMC*	2p23.3	High-fat high-sucrose diet in ontogeny associated with *POMC* promoter hypomethylation in POMC neurons in hypothalamus, and glucose response. Decreased acetylation of specific CpG *POMC* in hypothalamus according to diet in ontogeny. Selective downregulation after high-fat diet in ontogeny in hypothalamus. Neonatal overfeeding led to MetS phenotype, hypermethylation of hypothalamic *POMC* promoter, and childhood obesity. Early life stress produces hypomethylation and deacetylation of hypothalamic POMC neurons. Neonatal hypermethylation of hypothalamic POMC neurons anteceded adult insulin resistance. Hypermethylation of the *POMC* promoter leading to obesity with leptin resistance	Plagemann et al., 2009; Kuehnen et al., 2012; Mahmood et al., 2013; Wang et al., 2014; Wu et al., 2014; Zhang et al., 2014; Cifani et al., 2015; Voisin et al., 2015; Zheng et al., 2015	4.2
*POR*	7q11.2	Negative		3.4
*PPARG*	3p25	*PPARG*-CG1 methylation was significantly higher in individuals with higher visceral fat mass. Selective downregulation after high-fat diet in ontogeny. Hepatic expression of *PPARG* correlated to changes in promoter methylation, and to plasma leptin and ghrelin levels. Heterozygosity differences in *PPARG* methylation in adipose tissue	Fujiki et al., 2009; Schwenk et al., 2013; Nilsson et al., 2014; Wang et al., 2014; Cifani et al., 2015; Drogan et al., 2015	3.1
*SLC6A2*	16q12.2	Negative		3.3
*TCF7L2*	10q25.3	CpG sites of *TCF7L2, FTO* with differential DNA methylation in T2DM islets. Heterozygosity differences in *TCF7L2* methylation in adipose tissue. Changes in *TCF7L2* methylation after palmitate diet	Dayeh et al., 2014; Hall et al., 2014; Nilsson et al., 2014	3.2
*TLR4*	9q33.1	Hypomethylation of four CpGs in *TLR4* first exon in obese, methylation levels correlated with BMI	Remely et al., 2014a	2.3
*TNFA*	6p21.3	Association observed between *TNFA* methylation and LDL/HDL ratio	Bollati et al., 2014	2.3
*TRIB1*	8q24.13	Methylation changes in *TRIB1* after exercise in T2DM relatives	Nitert et al., 2012	3.1

*Description of meta-analytic method: To search for empirical studies in PubMed, gene locus, and keywords “methylation (hyper-, hypo-, de-),” “acetylation,” “phosphorylation,” “ubiquitination” and “epigenetics” and “metabolic syndrome” were entered. Included are only positive, but not negative results published until December 2015. “Negative” means that no specific research results pertaining to the metabolic syndrome were available*.

### Gene regulation

Further on were the results of microRNA studies in relation to MetS relevant processes evaluated. MicroRNAs are small non-coding RNA protein pieces that function as post-transcriptional gene regulators. Although transcription factors work both as activators and repressors, however, all currently known microRNAs solely work as repressors (Chen and Rajewsky, 2007) to protein translation.

To date, about 40 microRNAs have been identified pertaining to MetS (Dehwah et al., 2012) and in lipoprotein metabolism, specifically in glucose uptake, insulin secretion and adipogenesis. It is assumed that these play central roles in diabetic complications and progression to chronic diabetes (Wegner et al., 2014). Here, miR-495, miR-432, and miR-376a at chromosome 14q32 expression were found critical for survival of islets in pancreatic ß-cells in T2DM sufferers (Kameswaran et al., 2014). Best studied are hitherto microRNAs during adipogenesis and obesity, where miR-146b regulates the proliferation of visceral pre-adipocytes and promote their differentiation, a target of Krüppel-like transcription factor KLF7 (Chen et al., 2014). MiR-27a and miR-27b abundance decreased PPARγ during adipogenesis of human pluripotent adipose-derived stem cells, thus counteracting PPARγ (Karbiener et al., 2009; Kim et al., 2010). MiR-122 decrease was described central in hepatic fatty-acid and cholesterol synthesis rate, by reducing hepatosteatosis (Esau et al., 2006), together with miR-223 in obesity (Kilic et al., 2015), also with miR-425, miR-126, miR-16, miR-634 (Li et al., 2015), miR-519d, miR-27 and miR-519d for target PPARγ (McGregor and Choi, 2011), miR-26 triglyceride accumulation (Song et al., 2014). MiR-370, miR-22, miR-758 also engaged in liver-adipocyte interaction (Flowers et al., 2013), furthermore miR-10b, miR-302a, miR-378, miR-613, miR-224 (Peng et al., 2013, 2014). Regarding to epigenetic effects, MiR-21, miR-17, miR-200, miR-221/222, miR-203 have been found altered by glucose, or polysaturated fatty acid diets (Palmer et al., 2014), hence suggesting programming effects across the life-span.

Much less well studied have been the three key neuroendocrine systems leptin, melanocortin, and NPY. Leptin uptake and insulin secretion were found controlled by miR-200a, miR-200b, and miR-429, which are related to obesity. Leptin treatment downregulating these miRNAs in hypothalamus increased leptin receptor and insulin receptor expression, also inducing weight loss and improving hepatic insulin responsiveness (Crépin et al., 2014). Leptin upregulation by miR-383, miR-384-3p, and miR-488 provided proper functioning of activity of pro-opiomelanocortin (POMC) neurons (Derghal et al., 2015). Then, inhibition of miR-375 in the intermediate lobe of the pituitary gland increased POMC secretion, whereas miR-375 overexpression down-regulated ACTH secretion stimulated by CRH (Zhang et al., 2013). Deletion of the microRNA-processing enzyme Dicer, modulated by nutrient availability in the hypothalamus, defective glucose metabolism, and alterations in the pituitary-adrenocortical axis, resulted in neurodegeneration of POMC-expressing neurons in early ontogeny (Schneeberger et al., 2012). Further to that, it was found that loss of miR-103 due to Dicer knock-out triggered activation of mammalian target of rapamycin (mTOR) pathway in the arcuate nucleus leading to imbalance in the levels of neuropeptide Y, will be resulting in severe hyperphagia (Vinnikov et al., 2014). MiR-7a expression was particularly prominent in the SFO, a circumventricular organ with ependymal bridges of the blood-brain barrier, as well as the suprachiasmatic, paraventricular, periventricular, supraoptic, dorsomedial and arcuate hypothalamic nuclei and constrained to neuropeptide Y/AgRP-containing-neurons located in the ventromedial aspect of the arcuate nucleus (Herzer et al., 2012).

## Summary and conclusion

Research in the past decade has considerably sharpened previous concepts on aetiopathology of the constellation of MetS. It has now become possible to encircle and narrow in key pathophysiological processes. At the level of causation, three domains, namely nutritional factors, environmental and psychological stress factors, and chronic low-grade inflammatory processes in visceral adipose tissue can be focussed on. Nutrient-induced enforcement of oxidative stress in subcellular organelles, mitochondria and endoplasmic reticula are likely to bias steroidogenesis and to cascade from oxidative stress to impaired glucose uptake and insulin resistance. The current state of findings also justifies the assumption that stress susceptibility mainly in the central HPA may be reinforced by 11β-HSD-1 action in visceral adipose and hepatic tissues.

Main findings of the present meta-analytic investigation on the effects of posttranslational modifications in specific risk gene loci support the notion that psychological stress and nutrient impact lead to genotype-environmental interactions that shape the MetS phenotype. Recent evidence derived from studies of DNA methylation, histone acetylation, and other epigenetic processes have been able to support the central role of POMC neuron population in the hypothalamus (mostly in the arcuate nucleus). Summarising the convergent brain structures involved in the stress physiology of MetS, presently available evidence suggests that the processing of environmental stress is performed by basolateral amygdaloid and possibly central amygdaloid nuclei, which trigger sympathoexcitation. Central regulation of hunger-satiation homeostasis can, according to studies evaluated, be assigned to the interplay of arcuate and paraventricular nuclei. Less well investigated are the roles of circumventricular and SFOs in biasing the sympathetic output in interplay with leptin, MC4R, and NPY systems.

Novel aetiopathological and treatment concepts could arise from the fact that HPA dysfunction in MetS could be originated at the synthesis of glucocorticoids in a second peripheral CRH system that may be active in adipose tissue and liver thus biasing the stress susceptibility of the central hypothalamic-pituitary regulation. Future research should further investigate interactions of risk genotypes, environmental factors, and epigenetic programming in the pathophysiology of MetS.

## Author contributions

Drafting manuscript EL, OC, AL; Revising the manuscript for important intellectual content EL, OC, AL.

### Conflict of interest statement

The authors declare that the research was conducted in the absence of any commercial or financial relationships that could be construed as a potential conflict of interest.
