# Corrigendum: Neuroendocrinological and Epigenetic Mechanisms Subserving Autonomic Imbalance and HPA Dysfunction in the Metabolic Syndrome

**DOI:** 10.3389/fnins.2021.678258

**Published:** 2021-04-13

**Authors:** Erwin Lemche, Oleg S. Chaban, Alexandra V. Lemche

**Affiliations:** ^1^Section of Cognitive Neuropsychiatry, Department of Psychosis Studies, Institute of Psychiatry, Psychology and Neuroscience, King's College London, London, United Kingdom; ^2^Section of Psychosomatic Medicine, Bogomolets National Medical University, Kiev, Ukraine; ^3^Department of Medical Science, Institute of Clinical Research, Berlin, Germany

**Keywords:** metabolic syndrome, sympathetic autonomic nervous system, stress neuropsychobiology, hypothalamic-pituitary adrenocortical axis, epigenetic programming, gene regulation, microRNA, pathophysiology

The title of the original article was changed from “Neuroendorine and Epigentic Mechanisms Subserving Autonomic Imbalance and HPA Dysfunction in the Metabolic Syndrome” to “Neuroendocrinological and Epigenetic Mechanisms Subserving Autonomic Imbalance and HPA Dysfunction in the Metabolic Syndrome.”

In the original article, there was an error.

Instead of “DNA methylation, histone acetylation” it was erroneously written “histone methylation, lysine acetylation.”

A correction has been made to *Summary and Conclusion, Paragraph 2*. The corrected paragraph is shown below.

Main findings of the present meta-analytic investigation on the effects of posttranslational modifications in specific risk gene loci support the notion that psychological stress and nutrient impact lead to genotype-environmental interactions that shape the MetS phenotype. Recent evidence derived from studies of DNA methylation, histone acetylation, and other epigenetic processes have been able to support the central role of POMC neuron population in the hypothalamus (mostly in the arcuate nucleus). Summarising the convergent brain structures involved in the stress physiology of MetS, presently available evidence suggests that the processing of environmental stress is performed by basolateral amygdaloid and possibly central amygdaloid nuclei, which trigger sympathoexcitation. Central regulation of hunger-satiation homeostasis can, according to studies evaluated, be assigned to the interplay of arcuate and paraventricular nuclei. Less well investigated are the roles of circumventricular and SFOs in biasing the sympathetic output in interplay with leptin, MC4R, and NPY systems.

The authors apologize for this error and state that this does not change the scientific conclusions of the article in any way. The original article has been updated.

